# Comparative Proteome-Wide Analysis of Bone Marrow Microenvironment of β-Thalassemia/Hemoglobin E

**DOI:** 10.3390/proteomes7010008

**Published:** 2019-02-23

**Authors:** Saranyoo Ponnikorn, Rungrawee Mongkolrob, Suwit Klongthalay, Sittiruk Roytrakul, Kitima Srisanga, Sumalee Tungpradabkul, Suradej Hongeng

**Affiliations:** 1 Chulabhorn International College of Medicine, Thammasat University Rangsit campus, Pathum Thani 12121, Thailand; scrmr303@gmail.com (R.M.); kitima_su@hotmail.com (K.S.); 2 Faculty of Medical Technology, Rangsit University, Pathum Thani 12000, Thailand; suwit_klong51@hotmail.com; 3 National Center for Genetic Engineering and Biotechnology (BIOTEC), Thailand Science Park, Pathum Thani 12121, Thailand; sittiruk@biotec.or.th; 4 Department of Biochemistry, Faculty of Science, Mahidol University, Bangkok 10400, Thailand; pexcotung@gmail.com; 5 Department of Pediatrics, Faculty of Medicine Ramathibodi Hospital, Mahidol University, Bangkok 10400, Thailand

**Keywords:** β-thalassemia/Hb E, bone marrow, ineffective erythropoiesis, proteomics, oxidative stress, Apolipoprotein D

## Abstract

β-thalassemia/Hb E is a global health issue, which is characterized by a range of clinical symptoms from a mild and asymptomatic anemia to severe disorders that require transfusions from infancy. Pathological mechanisms of the disease involve the excess of unmatched alpha globin and iron overload, leading to ineffective erythropoiesis and ultimately to the premature death of erythroid precursors in bone marrow (BM) and peripheral organs. However, it is unclear as to how BM microenvironment factors contribute to the defective erythropoiesis in β-thalassemia/Hb E patients. Here, we employed mass spectrometry-based comparative proteomics to analyze BM plasma that was collected from six β-thalassemia/Hb E patients and four healthy donors. We identified that the differentially expressed proteins are enriched in secretory or exosome-associated proteins, many of which have putative functions in the oxidative stress response. Using Western blot assay, we confirmed that atypical lipoprotein, Apolipoprotein D (APOD), belonging to the Lipocalin transporter superfamily, was significantly decreased in BM plasma of the tested pediatric β-thalassemia/Hb E patients. Our results highlight that the disease condition of ineffective erythropoiesis and oxidative stress found in BM microenvironment of β-thalassemia/Hb E patients is associated with the impaired expression of APOD protein.

## 1. Introduction

Thalassemia is a heterogeneous group of anemias that involve a defective synthesis of mature hemoglobin (Hb). The pathophysiology of β-thalassemia/Hb E is strongly associated with the excess of unmatched α-globin chain and the mutation of hemoglobin variants [1]. These phenomena are believed to be the etiological factors of an ineffective erythropoiesis in BM causing hemolysis in peripheral circulation [2,3]. Patients with the β-thalassemia/Hb E genotype suffer from various clinical symptoms including anemia, splenomegaly, hepatomegaly, jaundice, abdominal mass, and fever [1,2]. These clinical manifestations are derived from chronic anemia and iron overload and are caused by an increased iron absorption in the gastrointestinal tract as well as long-term blood transfusions [3,4]. However, transfusion-independent thalassemia also shows iron overload due to peripheral hemolysis and ineffective erythropoiesis [3,5,6]. 

Pathological features at the cellular level of thalassemia include abnormal differentiation of erythroid progenitors to mature red cells, hyper-proliferation in BM, and increased cell death of erythroid precursors [7,8,9]. Both iron overload and unmatched alpha-globin are the key factors that lead to high oxidative stress in β-thalassemia patients [10]. Furthermore, the oxidative stress that was found during thalassemia erythroid cell development may be linked to the induction of apoptosis [11,12]. Some studies proposed relationships between apoptosis and oxidative stress of erythroid cells in β-thalassemia/Hb E, an analysis of erythroid lineage cells revealed a high abundance of apoptosis-related proteins, such as cytochrome C, caspase 6, and apoptosis inducing factors [13]. BM microenvironment influences regeneration and erythropoiesis activities of hematopoietic stem cells (HSCs) [14,15,16]. Heterogeneous regulatory factors, cytokines, signaling molecules, and various cellular interactions play important roles in the self-renewal, proliferation, differentiation, and mobilization of HSCs. However, the pathological BM microenvironment of β-thalassemia/HbE is still unknown. 

Mass spectrometry (MS) is a powerful high-throughput technology for investigating proteome-wide protein expression. This technology allows for in-depth analysis of limited amounts of human samples for molecular diagnosis. The MS-based method has been applied to analyze thalassemia-specific proteomes from HSCs and other tissues in a range of patient specimens [17,18]. In the current study, we aim to comprehensively understand the early molecular basis of β-thalassemia/Hb E, thereby comparing the protein expression levels of BM plasma samples of β-thalassemia/Hb E patients, which represent bona fide microenvironment of ineffective erythropoiesis. We report an interesting imbalance protein expression involving oxidative and anti-oxidative processes, which may contribute to ineffective erythropoiesis that is found in β-thalassemia/Hb E patients. 

## 2. Materials and Methods

### 2.1. Bone Marrow Samples

Bone marrow samples were received from six β-thalassemia/Hb E patients that admitted to Ramathibodi hospital, Bangkok, Thailand, and four normal donors. All patients were 3–10 years old and had clinical manifestation of severe anemia and hepatosplenomegaly. Twenty milliliters (mL) of bone marrow were aspirated from posterior iliac crest into syringe containing 0.1 mL of heparin anticoagulant and transported to the laboratory for the isolation of stem cells and obtaining of bone marrow supernatant. Sample collection was approved by Ethical Committee of Research on Human beings at Ramathibodi Hospital, Faculty of Medicine, Mahidol University, Bangkok and Thammasat University, Pathum Thani, Thailand (protocol number ID125306). The bone marrow supernatant was harvested by centrifugation of bone marrow aspirate at 3000 rpm for 5 min and stored at −80 °C for further analysis.

### 2.2. Protein Fractionation by One-Dimensional SDS-PAGE

All of the acetone precipitated samples and BSA (Sigma-Aldrich, St. Louis, MO, USA) were prepared in a final volume of 10 μL. The 190 μL of Bradford reagent (Bio-Rad, Hercules, CA, USA) was added to each well of a microtiter plate and carefully mix. The optical density (OD) of samples was read at a wavelength of 595 nm after 5 min incubation at room temperature. The concentration of sample was calculated using standard curve. The 12% gel SDS-PAGE was performed to separate the protein samples. Protein band quantitation and qualification were evaluated after the silver staining protocol, briefly gel was sensitized by adding 0.02% (*w*/*v*) Na_2_S_2_O_3_ for 2 min, then washed twice with milliQ water, and stained with 0.2% (*w*/*v*) AgNO_3_ for 20 min. After washing the gel with H_2_O for 30 s, color developing solution (6% (*w*/*v*) Na_2_CO_3_ 0.005% (*w*/*v*) Na_2_S_2_O_3_, 0.0185% (*v*/*v*) formaldehyde) were added to the gel until the desired protein bands presented. Stained gel was stopped using the 1.46% (*w*/*v*) EDTA and then stored in 0.1% acetic acid prior to finally observed the protein band using the GS-710 scanner (Bio-Rad, Hercules, CA, USA).

### 2.3. In-Gel Digestion of Proteins for LC-MS/MS

The protein bands from SDS-PAGE were excised into 1 × 1 × 1 mm^3^ sized gel plugs. The step of in-gel digestion was performed according to the previous description [18,19] with some modifications. Briefly, the gel plugs were placed into 96-well plate and washed with 200 μL/well of sterile milliQ water by shaking for 5 min at room temperature. After removing sterile milliQ water, the gel plugs were dehydrated twice with 200 μL/well of 100% acetonitrile (JT Baker) by shaking for 5 min at room temperature and the gel plugs were allowed to dry at room temperature for 10 min. The disulfide bonds of the protein samples were reduced by the addition of 50 μL/well of 10 mM dithiothreitol (DTT) in 10 mM ammonium bicarbonate (NH_4_HCO_3_) for 1 h at room temperature. After removing the reducing agent, 50 μL/well of 100 mM iodoacetamide (IAA) in 10 mM NH_4_HCO_3_ were added to prevent the reformation of disulfide bond and then incubation at room temperature for 1 h in a dark place. The protein samples were washed twice with 200 μL/well of 100% acetonitrile by shaking for 5 min at room temperature and then digested with 20 μL/well of 10 ng trypsin (Promega, Madison, WI, USA) in 50% acetonitrile/10 mM NH_4_HCO_3_ followed by incubation at room temperature for 20 min. The 30 μL/well of 30% acetonitrile were added and further incubated into the samples overnight at room temperature. The solutions containing peptide in each well were transferred into new low binding 96-well plate. The remaining tryptic peptides were extracted twice with 30 μL/well of 50% acetonitrile in 0.1% formic acid by agitation for 10 min at room temperature and then transferred to previous digestion solution. The gel pieces were finally extracted by the addition of 50 μL/well of 70% acetonitrile in 0.1% formic acid at room temperature for 10 min and transferred to the original digestion solution. Finally, the tryptic peptide solutions were dried by incubation at 40 °C for overnight and then kept at −80 °C until LC-MS/MS analysis.

### 2.4. LC-MS/MS Analysis

The dried peptide samples were twice dissolved with 15 μL/well of 0.1% formic acid, centrifuged at 12,000 rpm for 5 min, and transferred to the vial for injection to liquid chromatography-tandem mass spectrometry (LC-MS/MS). The samples were injected to LC-MS/MS system that was described in previous studies [19,20]. Briefly, the peptides were separated using a Famos-Switchos-Ultimate nano-LC system (Dionex, LC packing) that was interfaced with a QSTAR XL (Applied Biosystems; MDS-Sciex, Foster, CA, USA) tandem ESI-QUAD-TOF MS. The trapped peptides that were captured in a C18 trapping cartridge (0.3 mm inner diameter × 5 mm; LC Packings) were eluted onto a C18 analytical column (0.075 mm inner diameter × 100 mm; packed in-house with 5-μm particle size packing material from Column Engineering). The samples were initially transferred with an aqueous 0.1% formic acid solution (mobile phase A) to the trap column with a flow rate of 15 μL/min for 1 min. The peptides were separated with a gradient of 15–50% mobile phase B in acetonitrile with 0.1% formic acid over 15 min at a flow rate of 600 nL/min, followed by a 3 min rinse with 80% of mobile phase B. The column temperature was maintained at 35 °C. The lock mass was delivered from the auxiliary pump of the NanoAcquity system with a constant flow rate of 500 nL/min with 200 fmol/μL of [Glu1] fibrinopeptide B that was delivered to the reference sprayer of the NanoLockSpray source of the mass spectrometer. All of the samples of tryptic peptides were analyzed by using a SYNAPTTM HDMS mass spectrometer (Waters Corp., Manchester, UK), which was operated in the V-mode of analysis with a resolution of at least 10,000 full-width half-maximum, using positive nanoelectrospray ion mode. The time-of-flight analyzer of the mass spectrometer was externally calibrated with [Glu1] fibrinopeptide B from *m*/*z* 50 to 1600 with acquisition lock mass being corrected using the monoisotopic mass of the doubly charged precursor of [Glu1] fibrinopeptide B. The reference sprayer was sampled with every 20 sec. Accurate mass LC-MS data were acquired with data direct acquisition mode. The energy trap was set at the collision energy of 6 V. In the transfer collision energy control, low energy was set at 4 V. The quadrupole mass analyzer was adjusted, such that ions from *m*/*z* 300 to 1800 were efficiently transmitted. The MS\MS survey was over range from 50 to 1990 Da and scan time was 0.5 sec.

### 2.5. Database Searching and Protein Quantitation and Identification

The MS/MS data were converted from MassLynx to ASCII format using DataBridge software and then the data were processed using DeCyder MS Differential Analysis software (DeCyder MS, GE Healthcare, Boston, MA, USA) to quantitate the protein expression based on peptide intensity, which are generated from two-dimensional (2D) signal intensity maps displaying *m*/*z* intensities versus time. Firstly, the ASCII files were imported to specify the optimal condition in the TOF mass analyzer with a crop retention time of 6.5 to 15 min. Secondly, the PepDetect module was used to detect the peptide and assign the charge state. The peptides were detected using typical peak width of 0.2 min, TOF resolution of 9000, charge state assignments from 1–10, LC peak shape tolerance of 20%, *m*/*z* shift tolerance of 0.1 u, *m*/*z* shape tolerance of 5.0%, remove peptides below S/N of 1.0, remove peptides of unspecified charge below S/N of 1.0, remove peptides with low quality LC peaks, and remove overlapping peptides. Finally, PepMatch module was used to match the peptides across different signal peptide intensities, resulting in quantitative comparison at *p*-value < 0.01. The data were exported as the mgf format with charge state to export of 1 to 3, before peak (% of width) of 10, after peak (% of width) of 10, and *m*/*z* tolerance (u) of 0.1. The mgf files were searched against the NCBInr database using MASCOT MS/MS Ion search engine (Matrix Science) for protein identification under 1% false discovery rate (FDR) determination. Database interrogation was: taxonomy (Homo sapiens); enzyme (trypsin); global modifications (carbamidomethyl); variable modifications (oxidation of methionine residues); mass values (monoisotopic); max missed cleavages (3); protein mass (unrestricted); peptide mass tolerance (±1.2 Da); MS/MS tolerance (±0.6 Da), peptide charge state (1+, 2+ and 3+); and, instrument (ESI-QUAD-TOF). Finally, the data consisting of protein name, GI number, *p*-value, peptide sequence, ID score, and signal intensity were exported into excel files. The protein intensity with an expression ratio ≥1.5-fold and ≤0.67-fold were concerned as an over-expressed and under-expressed protein, respectively.

### 2.6. Bioinformatic Analysis

The UniProt Knowledgebase (UniProtKB; http://www.uniprot.org/) was used to collect the cellular compartment, functional information, including biological process, and molecular function of the protein. Briefly, the GI numbers from the final MS/MS data were searched against UniProtKB to access the protein information. UniProtKB ID was accessed to enter the protein information, including names and origin; protein attributes; general annotation; ontologies consist of keywords, gene ontology (biological process, cellular component, molecular function); sequence annotation; cross-references consist of three-dimensional (3D) structure databases, Protein-protein interaction databases, PTM databases, etc. The secretory protein classification was categorized as extracellular proteins identification by UniProtKB, and analytically processed under SignalP 4.1 and Secretome P2.0 server regarding the presence and location of signal peptide in amino acid. The classification of gene ontology was searched against the Protein Analysis through Evolutionary Relationships (PANTHER) classification system (http://www.pantherdb.org/). This software was designed to categorize the proteins according to their family and sub-family, molecular function, biological process, and pathway. The protein-protein interaction prediction from the proteome data was constructed and then executed using STRING (Search Tool for the Retrieval of Interacting Genes/Proteins; http://string-db.org/) 11.0. The evidence mode was set up with a medium confidence level at 0.4 and other search parameters are included: text mining, experiments, databases, co-expression, neighborhood, gene fusion, and co-occurrence. The protein–protein interaction network was analyzed from a collection of manually drawn pathway maps while using the KEGG (Kyoto Encyclopedia of Genes and Genomes) PATHWAY database. The MeV multi experiment viewer was selected to the Heatmap generation from the statistic significant of differential expression protein profiles.

### 2.7. Validation of Candidate Protein Expression Using Western Blot

The proteins from six available bone marrow supernatants from the same analyzed proteome samples included three patients and donors, respectively, were heated for 10 min at 100 °C before being loaded 10 μL onto 15% SDS-polyacrylamide gel in each lane of 25 μg protein with a pre-stained protein ladder (Vivantis, Selangor Darul Jaya, Malaysia). Electrophoresis had done for 1.50 h at 150 volt and then transfer the protein to polyvinylidene fluoride membrane (PVDF, GE healthcare, Boston, MA, USA) for 1 h at 300 mA. Membranes were blocked with blocking buffer (5% skim milk powder in TBST (Tris Buffered Saline with TWEEN 20) for 1 h at room temperature and then incubated with 1:1000 anti-human ApoD (Millipore, Burlington, MA, USA) in TBST overnight at 4 °C. Consequently, incubate membranes with 1:10,000 horseradish peroxidase conjugated anti-rabbit IgG (Cell signaling, Danvers, MA, USA) in TBST for 1 h at room temperature. Finally, apply the chemiluminescent blotting detection reagent (GE healthcare, Boston, MA, USA) and then incubate for 5 min at room temperature in the dark. The chemiluminescent signal was analyzed with camera-based imager (Amersham^TM^ Imager 600, GE healthcare, Boston, MA, USA). The equal loading control was done using Coomassie Brilliant Blue staining of a similar SDS-PAGE condition. Each protein band at 75 kDa calculated the intensity of dye using quantitative OD measurement from the colorimetric white transillumination option.

### 2.8. Statistical Evaluation

The Mann–Whitney–Wilcoxon test was used to compare the protein intensity values that were determined from 2D signal intensity maps in Decyder MS software (DeCyder MS, GE Healthcare, Boston, MA, USA) with significant statistical evaluation of a differential protein expression profile, *p*-value < 0.05 using MeV software analysis. Data was analyzed to each independent sample, including six patients and four donors. The comparative ApoD expression from DeCyder MS/MS data quantitation was analyzed with paired T-test at *p*-value < 0.01 using Prism version 7.

## 3. Results

### 3.1. Protein Identification, Classification and Categorization of the Bone Marrow Microenvironment Protein Profile from β-Thalassemia/Hb E

The BM plasma samples were collected from six β-thalassemia/Hb E patients and four healthy donors. Total plasma proteins were precipitated, fractionated in one-dimensional SDS-PAGE, and visualized by mass spectrometry-compatible silver staining. We observed differential expression of several protein bands between patients and healthy donors (Figure S1). In-gel digestion of excised gel pieces was performed along with standard molecular weight markers. Peptide sequence identification was performed using ESI-QTOF-MS/MS and differential protein expression levels of *p*-value < 0.01 were identified using DeCyder MS Differential Analysis Software and BioTools. We found 392 proteins that were differentially expressed in β-thalassemia/HbE samples (Figure S2 and Table S1). 

**Figure 1:**
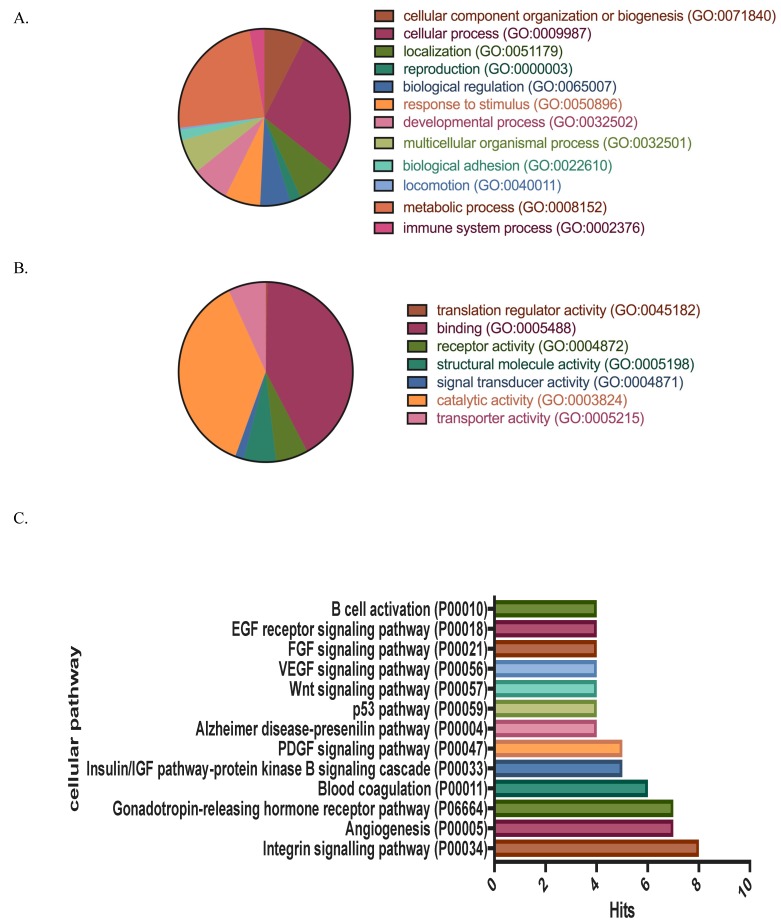
The functional analysis of (**A**) biological process, (**B**) molecular function, and (**C**) cellular pathway of identified protein from β-thalassemia/Hb E and healthy donors.

Gene ontology analysis that was based on PantherDB and STRING database was used to assign biological processes and molecular functions of the differentially expressed proteins. Proteins were classified and categorized by biological processes as cellular process (GO:0009987) and metabolic process (GO:0008152). The predicted molecular functions of the protein set include binding (GO:0005488) and catalytic activities (GO:00003824) (Figure 1A,B). To obtain relevant biological and biochemical pathways that are associated with the identified proteins, we used the cellular pathway module from Panther analysis. These proteins were then categorized and classified together with UniProt gene ontology to match with cellular pathways by various relationships, such as protein interactions, modifications, and the regulation of expressions. We show that several biochemical pathways are altered in the BM samples of β-thalassemia/Hb E. These predictions allowed for further identification of candidate proteins with specific relevance to the β-thalassemia/HbE condition.

**Figure 2:**
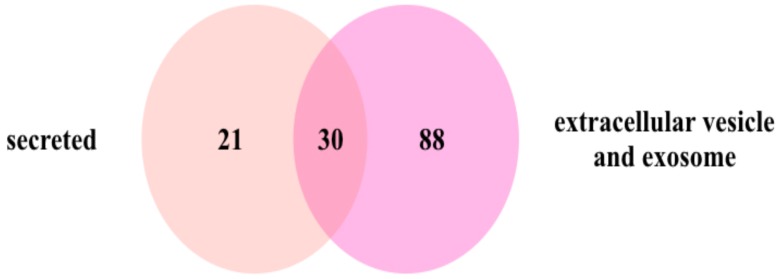
The Venn diagram represents the classification and categorization of the extracellular vesicles and secretory proteins in the proteome.

The cellular pathways included Integrin signaling pathway (P00034), Angiogenesis (P00005), Gonadotropin-releasing hormone receptor pathway (P06664), Blood coagulation (P00011), Insulin/IGF pathway-protein kinase B signaling cascade (P00033), and PDGF signaling pathway (P00047) (Figure 1C). The UniProtKB, SignalP 4.1, and Secretome P2.0 server categorized the 106 identified proteins as extracellular proteins. The classification and categorization of these proteins are represented in a Venn diagram (Figure 2). Most of the proteins have overlapping categories between extracellular vesicles and extracellular exosomes from the database. Interestingly, 51 proteins are characterized and identified the signal sequence that determines the secretory proteins (Figure 2 and Table S2).

**Figure 3:**
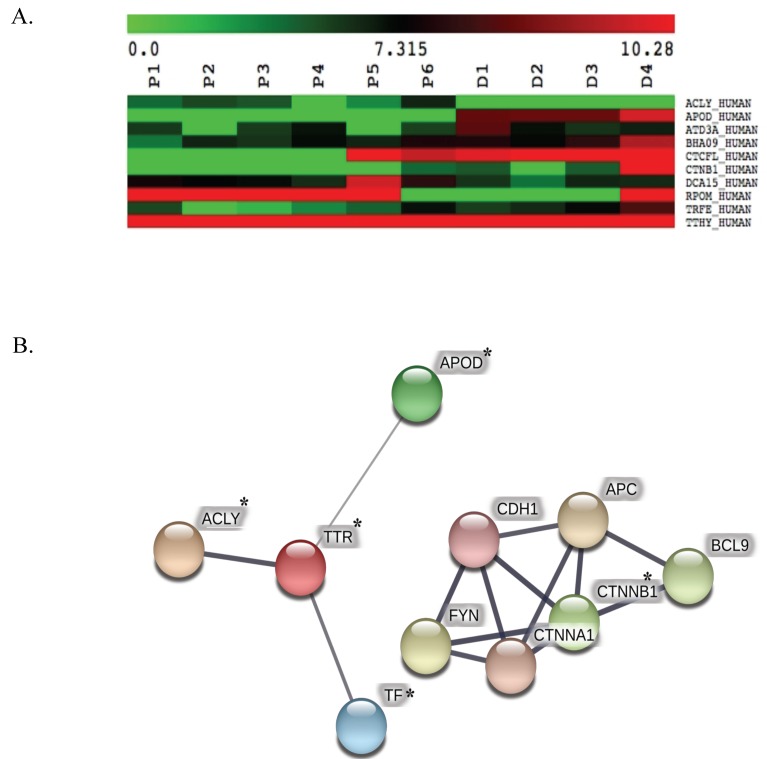
The visualization of expression level of 10 proteins and their protein-protein interaction networks (PPIs) from the significant differential expressed protein identification. Heatmap of relatively high (red) and low (green) proteins from six patients (P) and four donors (D), colour gradient represent protein intensity values determined from two-dimensional (2D) signal intensity maps in DeCyder mass spectrometry (MS) software. The heatmap were generated the significant expression protein using Wilcoxon at *p*-value <0.05 (**A**). The protein-protein interaction networks of the significant candidate proteins were constructed using Search Tool for the Retrieval of Interacting Genes/Proteins (STRING), protein nodes with asterisk (*) are the identified proteins in this study (**B**).

The 392 differentially expressed proteins were analyzed. The degree of protein expression among patients and normal donors using the Mann–Whitney–Wilcoxon test (*p*-value ≤ 0.05) are represented using a heatmap visualization (Figure 3A). Ten proteins were identified as statistically significant with differential expression in six patients and four donors. STRING 11.0 carried out the protein–protein interaction networks (PPIs). The PPIs were constructed under an increasing number of node proteins equal to 20 with a local clustering coefficient degree at 0.53. The visualization of PPIs is represented by the cellular pathway analysis under the KEGG database of PPIs enrichment at a *p*-value < 0.001 (Figure 3B, Table 1, Table S3). Based on MS quantification between patients and donors, five proteins that were under-expressed in β-thalassemia/HbE include Apolipoprotein D (APOD_HUMAN), ATPase family AAA domain-containing protein 3A (ATD3A_HUMAN), Transcriptional repressor CTCFL (CTCFL_HUMAN), Catenin beta-1(CTNB1_HUMAN), and Serotransferrin (TRFE_HUMAN). Two proteins are over-expressed in β-thalassemia/HbE, including ATP citrate lyase (ACLY_HUMAN) and mitochondrial DNA-directed RNA polymerase (RPOM_HUMAN).

### 3.2. ApoD Protein and Validation of the Proteome

**Figure 4:**
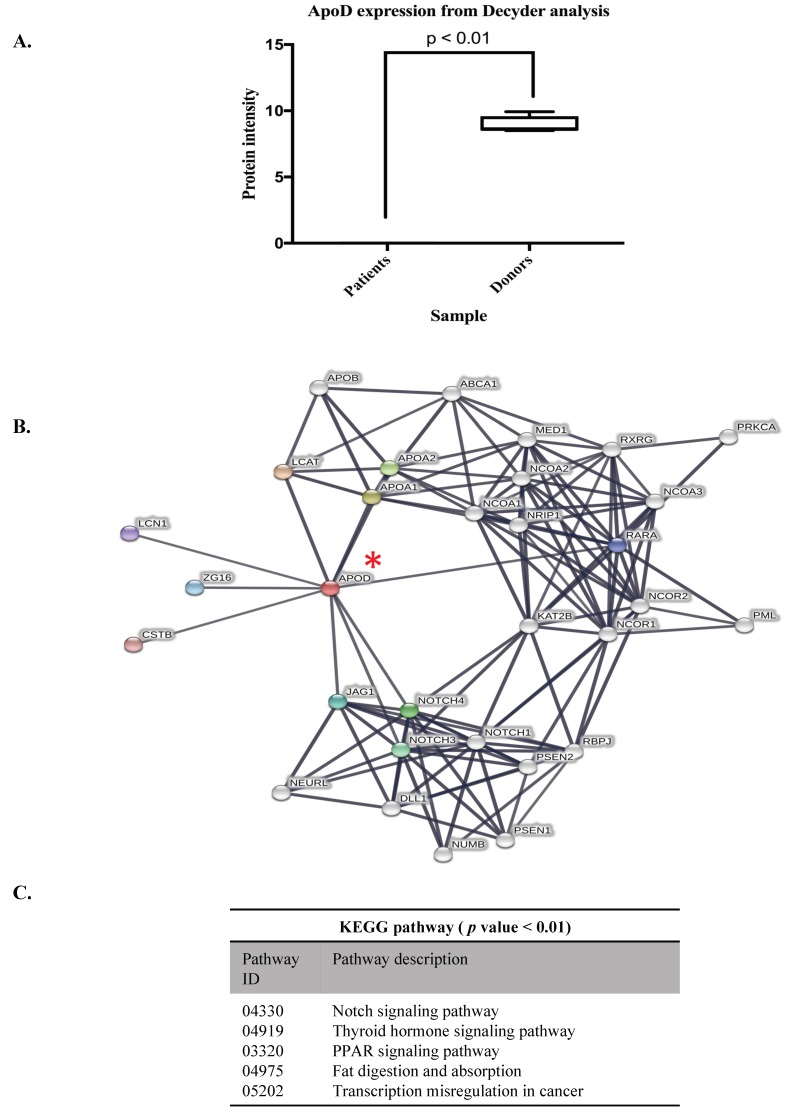
The visualization of protein-protein interaction networks (PPI) of Apolipoprotein D. The DeCyder analysis of MS/MS spectra comparison of Apolipoprotein D (APOD) expression between patients and donors with significant relative expression level at p value < 0.01 (**A**). The protein-protein interaction network of APOD (red asterisk) was constructed from STRING 11.0 (**B**), then using KEGG pathway analysis of this PPI revealed five relevant signaling pathways of APOD interactome (**C**).

Various proteins in the bone marrow microenvironment dynamically orchestrate erythropoiesis. The ineffective erythropoiesis in β-thalassemia has been strongly associated with the increase of oxidative stress in erythroid progenitor cells. However, the interaction between the elevated ROS in erythroid progenitor cells and the pathological of bone marrow tissue has not been addressed. Here, the bone marrow plasma proteome identified a significant under-expressed protein involving oxidative damage, Apolipoprotein D. An atypical lipoprotein, Apolipoprotein D (APOD), was significantly under-expressed in β-thalassemia BM plasma samples based on our mass spectrometry data (Figure 4A and Figure S3). The APOD belongs to the lipocalin family that was responsible for binding to small hydrophobic molecules, such as bilirubin, arachidonic acid, progesterone, and cholesterol. The APOD is also classified and categorized as exosomal protein from cancer cells under the KEGG database (Table 1). However, there are very limited reports regarding the functions of APOD in hematopoiesis, and especially in thalassemia. 

**Figure 5:**
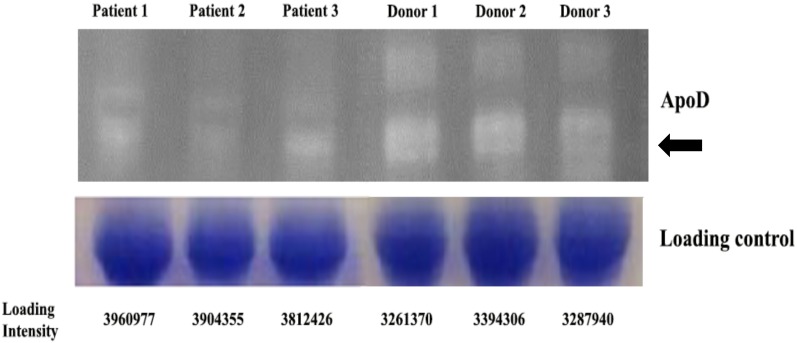
The validation of Apolipoprotein D expression by western blot in β-thalassemia/HbE marrow plasma protein from patients and normal donors. The validation of the proteomic analysis of human bone marrow plasma was investigated the APOD expression of bone marrow plasma isolated from three β-thalassemia/HbE patients and three donors. The band of APOD protein is approximately 29–30 kDa (arrow). gel with Coomassie staining for determining the equal loading control was compared with each protein samples and calculated the quantitative optical density (OD) measurement at 75 kDa.

To construct protein–protein interaction networks (PPIs) of APOD and related neighboring candidate partner proteins, the STRING 11.0 was used to analyze and predict the relevant biochemical and signaling pathways under protein–protein interaction mapping. The PPIs of APOD are visualized under an increasing number of node protein equal to 41 with local clustering coefficient degree at 0.689 and they are represented with the cellular pathway analysis under the KEGG database as PPIs enrichment at a *p*-value < 0.002 (Figure 4B,C). Interestingly, five KEGG pathways are predicted as APOD interactome, including NOTCH signaling pathway (04330), thyroid signaling pathway (04919), PPAR signaling pathway (03320), fat digestion and absorption (04975), and transcriptional misregulation in cancer (05202) (Figure 5C). This analysis revealed APOD is involved in various molecular functions and cellular pathways. However, the molecular mechanism of APOD in ineffective erythropoiesis in thalassemic disease has not been elucidated.

Finally, the APOD protein expression was validated using western blot analysis. APOD expression is consistently within the DeCyder MS analysis of the proteome; the protein expression of APOD from all patients was significantly lower than the healthy donors (Figure 5).

## 4. Discussion

Ineffective erythropoiesis is a pathophysiological condition in β-thalassemia, which is characterized by abnormal erythroid cell expansion and differentiation and destruction of premature erythroid precursors in bone marrow [1]. In normal conditions, erythropoiesis is regulated by both microenvironment and signaling factors inside BM, involving sequential and specific erythroid gene regulation [21]. However, how these factors contribute to the abnormal erythropoiesis in β-thalassemia is poorly understood. Our study is the first report using the label-free quantitation proteomics to analyze the limited human BM samples that represent the microenvironment of hematopoietic stem cells after protein enrichment or separation procedures as previous studies [13,20]. Ten significantly differentially expressed proteins were selected for the construction of PPIs using STRING 11.0 prediction and the KEGG pathway. We further validated under expression of the apolipoprotein D (APOD_HUMAN) in the patient samples using western blot analysis.

The atypical lipoprotein APOD belongs to the lipocalin family that is responsible for the binding of small hydrophobic molecules, such as bilirubin, arachidonic acid, progesterone, and cholesterol. The APOD is found in plasma and various human tissues, especially in the brain [22,23]. Interestingly, our proteomic study discovered the lower expression of APOD in human BM plasma. However, this protein has not been studied for its relevant function in the pathophysiology of thalassemia. Many previous reports demonstrated the function of APOD in various physiological and pathological states, including oxidative stress responses, lipid metabolism, molecular regulation in cancer, and aging [24,25,26]. 

Oxidative stress in β-thalassemia has become a hallmark of molecular pathological consequence that is mediated by the alpha excess and iron overload, which involve many destructive mechanisms at a cellular level to many organ failures in β-thalassemia patients [11,12]. The highly dynamic reactive oxygen species (ROS) level controls the regulation of growth, development, and differentiation of hematopoietic stem cells in bone marrow niche. The protection of a high ROS level is critical in preventing stem cell exhaustion, insufficient host immunity, and leukemic transformation [20]. In β-thalassemia patients, there is a significant increase in ROS production both in early and late erythroid precursors in erythroblast. This results in cellular damage and reduced life span of erythroid precursors [27]. However, the excessive ROS levels and increased apoptosis in erythroid precursors of β-thalassemia has not been well addressed. The regulation of APOD in the tissue microenvironment where ROS are elevated has been distinctly clarified in the experiments of mouse brain [23,28,29,30]. In a mouse model, the lack of APOD caused increased stress susceptibility, while APOD overdose rendered the animal less sensitive to the same stresses [23,30]. Moreover, the reduced APOD expression is found to be associated with increased levels of lipid peroxides in brain tissues [30]. Whereas, in humans, plasma and the brain are the main production sites of APOD. APOD is expressed by glia and other non-neuronal cells, which profoundly affects the function and the survival of neurons by disrupting the extracellular lipid transport, AA metabolism in an antioxidant, and anti-inflammatory function [31,32]. In addition, iron overload in thalassemia causes dyslipoproteinemia. This action is mediated by the lipid peroxidation, which is demonstrated as the existence of oxidized HDL with poor ability to protect LDL from oxidation in thalassemia. The diminished protective of HDL is consistent with the compositional changes and the high level of oxidative markers [33]. The prognostic biochemical cardiovascular risk factors in patients with β-thalassemia revealed a significantly lower apolipoprotein and lipid profile when compared with control individual [34]. Moreover, a significant reduction of APOD level had been reported in Tangier disease, which contributes the excessive accumulation of cholesterol in the body, and thereby increases the risk of developing atherosclerosis [24,35]. Suggesting the roles of lipid metabolism and fat digestion and absorption could help in determining the KEGG pathway in this study. Here, the possible correlation of a reduced form of expressed APOD in β-thalassemia bone marrow plasma, with relevant functions of APOD in lipid metabolism, could be related to the dyslipidemia and other complications in patients. In our study, the decreased APOD expression in the bone marrow of β-thalassemia/HbE might contribute to the excessive oxidative milieu during erythropoiesis. In normal condition, APOD is localized to the Golgi apparatus where protein modification takes place and is secreted, followed by the reinternalization of APOD into the cytoplasm. Nuclear accumulation of APOD could be observed under certain stress conditions. Interestingly, the addition of exogenous APOD caused an increased cell proliferation via an internalization of APOD to the cell, whereas apoptosis was observed when APOD was added to the growth-arrested cells. These suggest the roles of APOD in the balance between apoptosis and cell proliferation [26,36].

Ineffective erythropoiesis in β-thalassemia is also characterized by erythroid hyperplasia. In the β-thalassemia/HbE condition, imbalanced homeostasis of signaling in erythroid cells drives the hyper-proliferation of pro-erythroblasts via elevated levels of extracellular signal-regulated kinase 1/2 (ERK1/2) phosphorylation, accompanied with the de-regulated cAMP and Ca^2+^ signaling [37]. Remarkably, the APOD protein also interacts with the mitogen-activated protein kinase (MAPK) signaling pathway in ovine vascular smooth muscle cells. The APOD inhibits platelet-derived growth factor (PDGF)-BB-induced proliferation via the prevention of nuclear translocation of ERK1/2 [38]. Therefore, the under-expression of APOD in BM of β-thalassemia/HbE patients could explain the deregulation of MAPK pathway, resulting in the hyper-proliferation of erythroid cells during ineffective erythropoiesis. In addition, other cell signaling pathways have been reported for deciphering the imbalance of ineffective erythropoiesis in β-thalassemia. Phosphoproteomic analysis of CD34 positive hematopoietic stem cells (HSCs) revealed the increased cell death signaling pathways that are possibly mediated by the imbalance of AKT and JNK during oxidative damage, which has consequences to the 14-3-3 and the FOXO3 functions [13]. The elevated ROS levels in β-thalassemia erythroid cells play an important role in the activation of AKT, potentially resulting in the repression of FOXO3 activity, and hence reducing the response to oxidative stress [39]. However, JNK has another modulation for FOXO3, which promotes its transcriptional activity inside the nucleus [40]. Interestingly, the JNK signaling regulates the transcriptional control of a vertebrate homologue of Apolipoprotein D in the *Drosophila* Lipocalin family member as Neural Lazarillo [NLaz]. Moreover, the loss of Nlaz function reduces stress resistance and lifespan, while its over-expression represses growth, promotes stress tolerance, and extends lifespan [41]. Additionally, APOD expression is triggered in astrocytes, downstream of the stress sensitive JNK pathway, and the secretory form of APOD provides autocrine protection in glial cell against paraquat-induced oxidative damage [29]. However, the associated function of APOD with these signaling molecules, including AKT, JNK, and FOXO3, has not been studied in thalassemia.

The BM microenvironment consists of various cell types, including mesenchymal stem cells, endothelial cells, and adipocyte cells, as well as other small molecules controlling erythropoiesis. Nonetheless, it remains unclear what is the original cellular resources of APOD regulating stem cell functions in BM milieu. Studies on endothelial cells revealed that the paracrine effect causes the partial down-regulation of APOD during endothelial and mural cells interaction. APOD expression within mural cells is modulated through secreted factors and a cell surface receptor NOTCH3 to facilitate the binding of mural cells to the vascular basement membrane, leading to a stable and mature blood vessel [42].

## 5. Conclusions

Here, we report that the pathophysiology of β-thalassemia involved alteration of the human APOD expression in BM microenvironment, based on mass spectrometry and Western blot analyses of BM plasma. The under-expression of human APOD could affect cellular stress responses and signal transduction cascades in the BM of β-thalassemia patients. More studies are needed to define the biological functions of APOD in the regulation of erythroid cells and other cellular components in BM. Therefore, we propose that the APOD is a potential candidate disease marker of β-thalassemia-related ineffective erythropoiesis.

## Figures and Tables

**Table 1:** Evaluation of biological processes and molecular function and relative protein expression of significant proteins from constructed protein-protein interaction network with Kyoto Encyclopedia of Genes and Genomes (KEGG) analysis.

Uniprot	Name	Relative Protein Expression *	KEGG Analysis
ACLY_HUMAN	ATP citrate lyase	over expression ^#^	TCA cycle
APOD_HUMAN	apolipoprotein D	under expression ^#^	Exosomal proteins of other cancer cells
ATD3A_HUMAN	ATPase family AAA domain-containing protein 3A	under expression^#^	Mitochondrial biogenesis
BHA09_HUMAN	Class A basic helix-loop-helix protein	0.785	-
CTCFL_HUMAN	Transcriptional repressor CTCFL	under expression ^#^	-
CTNB1_HUMAN	Catenin beta-1	under expression ^#^	^1^ Wnt signaling pathway
			^2^ Hippo signaling pathway
			^3^ Signaling pathways regulating pluripotency of stem cells
DCA15_HUMAN	DDB1- and CUL4-associated factor 15	1.368	Ubiquitin system
RPOM_HUMAN	DNA-directed RNA polymerase, mitochondrial	over expression ^#^	Mitochondrial biogenesis
TRFE_HUMAN	Serotransferrin	under expression^#^	^1^ HIF-1 signaling pathway
			^2^ Ferroptosis
			^3^ Mineral absorption
TTHY_HUMAN	Transthyretin	1.002	Thyroid hormone synthesis

Relative protein expression (*) is determined by the mean protein intensity of patients divided by the mean protein intensity of donors. # relative protein expression > 1.5 and < 0.67 are classified as the over-expressed and under-expressed proteins, respectively.

## Supplementary Materials

The following are available online at https://www.mdpi.com/2227-7382/7/1/8/s1, Figure S1: SDS-PAGE fractionation with silver staining of bone marrow supernatant from six β- thalassemia/Hb E patients (P) and four donors (D), Figure S2: The heatmap visualization of 392 proteins from six β- thalassemia/Hb E patients (P) and four donors (D), Table S1: The 392 proteins with MS identified and Uniprot ID from six β- thalassemia/Hb E patients (P) and four donors (D), Table S2: The 106 proteins determining the secretory and exosomal protein characterization in this study, Table S3: The 10 significant expressed proteins with analytical data, Figure S3: A MS/MS fragmentation of Apolipoprotein D.

## Abbreviations

β-thalassemia/HbE	beta thalassemia with variant hemoglobin E
ESI-QTOF-MS/MS	electrospray-ionisation-quadrupole-time-of-flight tandem mass spectrometry
STRING	Search Tool for the Retrieval of Interacting Genes
PPIs	protein-protein interaction networks
APOD	Apolipoprotein D
HSCs	hematopoietic stem cells
