# Supplementary material for: Comparative Proteome-Wide Analysis of Bone Marrow Microenvironment of β-Thalassemia/Hemoglobin E
Source: Proteomes. 2019 Feb 23;7(1):8. doi: 10.3390/proteomes7010008 (PMC6473223; doi:10.3390/proteomes7010008)
Supplement: Supplementary file 1 [file proteomes-07-00008-s001.pdf]

Figure S1

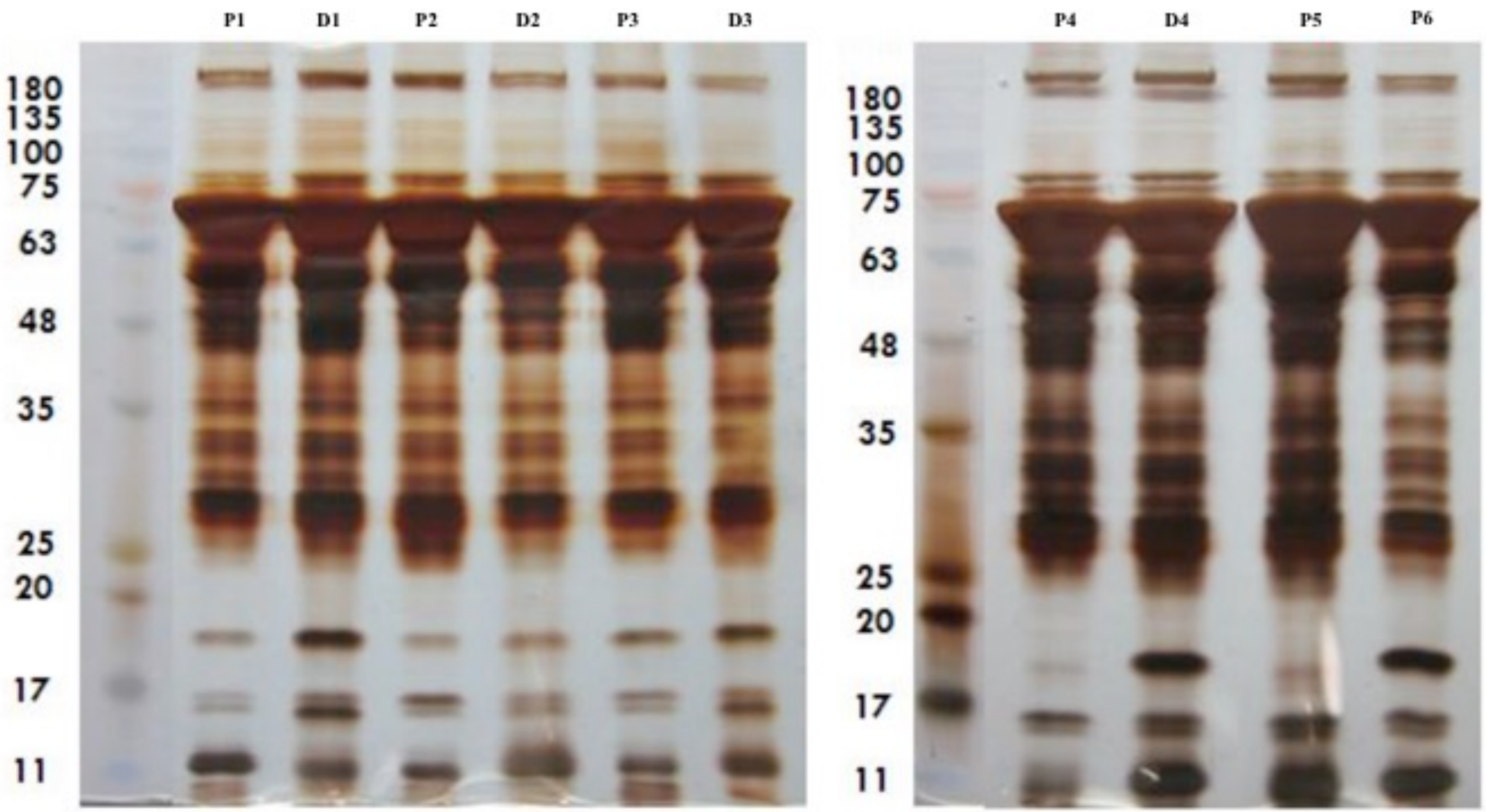

Additional file 1: SDS-PAGE fractionation with silver staining of bone marrow supernatant from six β- thalassemia/Hb E patients (P) and four donors (D).

Figure S2

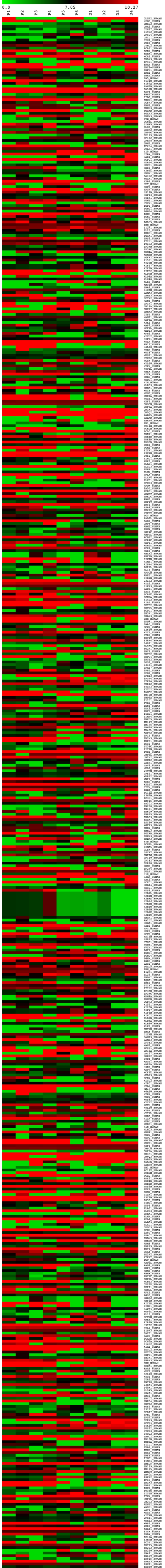
0.0
7.05
10.27
P1
P2
P3
P4
P5
P6
D1
D2
D3
D4

Table S1

The 392 proteins with MS identified and Uniprot ID from 6 patients (P) and 4 donors (D)

Uniprot KB	P1	P2	P3	P4	P5	P6	D1	D2	D3	D4
CU025_HUMAN	6.53	5.98	5.59	6.25	7.38	8.09	6.8	5.81	7.81	6.94
CUL3_HUMAN	0	0	7.6	3.56	5.69	0	11.01	0	0	0
CUL4B_HUMAN	8.61	8.11	8.56	9.46	9.54	7.4	9.26	9	9.57	10.67
CUX2_HUMAN	1.8	6.51	2.99	5.02	2.29	1.98	2.74	2.61	2.11	2.8
CWC22_HUMAN	8.36	8.89	8.05	8.62	8.13	8.77	9.12	8.74	8.06	8.59
CYB5_HUMAN	5.78	9.72	5.26	9.47	0	5.77	0	9.68	0	10
D19L2_HUMAN	0	0	0	0	4.94	0	6.18	0	0	8.56
DAZP2_HUMAN	0	7.1	3.54	3.88	0	0	3.79	3.96	5.3	8.73
DCA15_HUMAN	7.53	7.25	7.02	6.11	9.86	7.84	5.82	3.91	6.34	6.15
DCD2C_HUMAN	5.71	6.39	5.29	6.49	8.37	9.08	5.91	6.57	7.8	0
DCLK2_HUMAN	9.4	9.82	8.48	10.6	0	0	9.76	8.47	0	0
DDX_HUMAN	9.65	9.54	9.63	9.79	9.78	9.82	9.68	9.78	9.96	9.86
DDX5_HUMAN	8.48	8.65	9.79	8.16	8.11	8.3	8.25	8.09	8.46	8.16
DGCR8_HUMAN	7.82	8.93	10.83	6.47	9.7	11.78	9.64	12.25	9.57	8.36
DHH_HUMAN	8.08	8.14	8.49	8.48	8.16	8.5	8.13	8.22	8.09	8.48
DHX30_HUMAN	7.53	6.29	6.37	5.6	0	0	10.08	6.27	0	0
DHX8_HUMAN	11.9	12.02	12.07	11.92	12.06	12.07	12.07	12.06	12.07	12.03
DLGP2_HUMAN	13.5	0	0	0	13.73	13.72	0	13.72	13.72	13.72
DMXL2_HUMAN	0	0	0	2.97	4.72	6.18	0	4.59	6.91	7.03
DNA2_HUMAN	5.51	4.76	3.47	3.31	3.68	4.53	3.67	5.95	4.11	6.19
DPOD3_HUMAN	0	8.42	0	0	0	8.19	0	0	0	8.86
DPYL4_HUMAN	6.11	4.67	3.91	7.93	6.53	9.88	7.66	2.81	5.1	4.63
DSCL1_HUMAN	0	0	0	7.25	0	7.07	0	0	0	7.87
DUS8_HUMAN	7.32	7.12	7.05	8.17	6.9	7.19	7.45	7.78	6.52	7.17
DYH17_HUMAN	6.25	0	5.34	0	4.32	7.65	6.05	0	8.18	6.64
DYHC2_HUMAN	8.11	8.75	8.59	8.13	9.05	3.99	8.31	8.05	4.05	8.13
DYST_HUMAN	0	6.62	0	0	5.81	6.03	5.96	7.02	6.29	7.96
EAA5_HUMAN	5.58	5.93	5.61	4.98	6.11	5.99	5.24	5.73	6.32	6.86
EAA5_HUMAN	5.58	5.93	5.61	4.98	6.11	5.99	5.24	5.73	6.32	6.86
ECE2_HUMAN	11.48	11.51	11.46	11.51	0	0	11.49	0	0	11.46
EDRF1_HUMAN	8.35	0	0	8.34	9.59	8.28	0	9.59	8.28	0
ENAH_HUMAN	11.43	11.44	11.44	0	11.71	11.71	11.47	11.69	11.68	11.69
ERC2-HUMAN	12.73	12.81	12.78	12.78	12.8	0	12.8	12.82	0	12.78
ERR1_HUMAN	9.35	7.34	7.97	9.33	6.38	4.97	9.4	7.23	5.82	5.31
ESPL1_HUMAN	8.21	4.47	8.7	0	0	0	6.79	0	0	9.64
F131C_HUMAN	13.44	13.36	13.39	13.42	0	0	13.41	13.45	0	13.45
F187A_HUMAN	7.17	9.82	5.37	7.45	6.16	7.98	6.5	6.14	7.6	6.67
FA5_HUMAN	0	8.27	7.48	8.61	5.78	6.02	2.9	7.03	7.49	5.6
FA50B_HUMAN	10.27	10.31	10.29	10.31	10.32	10.32	10.28	10.53	10.52	10.53
FAM3B_HUMAN	0	0	0	0	8.12	8.16	0	8.12	8.16	8.18
FAT4_HUMAN	11.87	11.86	11.86	11.87	11.89	11.9	11.86	11.88	11.88	11.87
FBLN3_HUMAN	3.92	5.35	0	0	8.52	6.48	0	0	4.9	6.61
FBN1_HUMAN	0	3.45	2.38	3.9	6.13	7.24	4.09	5.1	4.72	4.43
FHOD1_HUMAN	10.96	11.01	11.01	11.02	11.02	11.04	11.01	11.01	12.01	11.01
FIBG_HUMAN	9.21	4.36	4.4	9.18	4.34	4.39	9.43	4.42	4.45	9.18
FMN2_HUMAN	8.26	8.33	8.3	7.97	8.3	0	8.32	8.33	8.33	7.97
FMNL3_HUMAN	8.21	0	8.18	8.14	8.22	8.64	0	8.56	8.2	8.56
FOCAD_HUMAN	12.98	13	13.38	12.99	12.92	0	12.98	12.92	0	0
FOXN3_HUMAN	5.94	0	5.99	6.17	6.35	4.99	0	0	7.64	8.13
FREM3_HUMAN	0	9.91	9.61	9.64	8.82	9.57	0	0	9.56	9.61
FYB_HUMAN	0	4.15	0	4.82	6.83	0	0	4.7	0	7.72
GCN1L_HUMAN	0	0	0	0	0	10.49	0	10.46	10.49	10.46
GIMA5_HUMAN	10.38	9.37	8.71	11.08	7.42	7.92	10.15	10.35	9.31	7.81
GLSK_HUMAN	8.59	8.87	6.93	10.18	0	0	9.15	6.92	0	0
GNPTG_HUMAN	9.29	8.51	9.03	9.11	8.45	9.39	8.7	8.45	9.35	8.83
GP119_HUMAN	0	0	0	0	3.07	6.71	0	0	2.89	4.12
GP162_HUMAN	0	0	8.42	8.41	8.23	8.03	0	8.31	8.31	8.42
GPR78_HUMAN	6.77	6.28	6.18	6.68	6.32	7.68	6.97	6.62	7.16	6.04
GRM5_HUMAN	0	8.72	0	0	0	0	8.65	0	8.55	7.35
GRP3_HUMAN	4.78	4.8	3.98	10.46	0	3.56	7.34	7.2	3.09	2.83
GSCR2_HUMAN	3.89	3.86	8.03	3.88	7.98	7.92	7.94	7.98	7.95	8
GTR6_HUMAN	7.76	5.97	8.08	8.72	6.47	7.16	5.47	7.66	5.85	4.65
GULP1_HUMAN	8.39	8.51	8.48	8.4	8.4	8.43	8.48	8.44	8.45	8.4
H10_HUMAN	4.88	4.86	4.87	4.88	10.7	10.71	4.86	10.7	10.71	5.1
H2AW_HUMAN	11.04	10.96	11.02	11	11	11	11	10.99	10.9	11
HAKAI_HUMAN	4.81	6.73	4.79	7.72	0	5.46	9.1	0	4.52	3.33
HAX1_HUMAN	0	0	0	0	6.01	0	0	4.49	5.59	10.54
HCFC1_HUMAN	13.3	13.32	13.33	14.69	13.35	13.32	13.31	13.32	13.34	14.69
HEAT4_HUMAN	0	0	0	0	5.91	6.76	0	0	0	8.78
HECD1_HUMAN	4.64	6.05	3.95	6.25	5.74	6.52	4.99	6.02	4.02	7.85
HEPS_HUMAN	0	4.71	0	6.12	6.84	8.53	0	0	8.5	8.13
HES6_HUMAN	9.06	9.08	8.85	10.9	0	0	9.69	8.11	0	0
HMGN1_HUMAN	5.89	7.94	5.45	6.44	5.65	6.03	6.09	5.4	6.04	6.27
HOGA1_HUMAN	7.82	7.34	6.28	9.3	0	0	8.37	5.75	0	0
HPT_HUMAN	9.56	0	0	9.57	9.61	0	0	0	0	9.57
HPTR_HUMAN	11.31	11.32	11.33	11.36	11.36	0	10.26	0	10.22	11.36

The 392 proteins with MS identified and Uniprot ID from 6 patients (P) and 4 donors (D)

Uniprot KB	P1	P2	P3	P4	P5	P6	D1	D2	D3	D4
HS12B_HUMAN	9.02	8.32	0	0	10.25	10.25	9.04	10.24	10.25	10.24
HSP13_HUMAN	6.96	6.43	4.1	7.32	7.53	7.47	7.13	7.52	7.43	8.76
HTSF1_HUMAN	6.59	5.57	5.57	5.58	6.87	6.34	6.12	6.09	5.81	5.36
HUWE1_HUMAN	9.75	9.85	9.03	9.77	9.11	8.67	10.34	9.51	8.37	8.36
HVCN1_HUMAN	0	10.17	10.44	10.18	0	0	0	0	0	10.44
HXB2_HUMAN	10.63	10.71	10.67	10.68	10.66	10.69	10.69	10.69	10.68	10.68
I12R1_HUMAN	8.7	8.51	8.53	8.55	9.03	10.16	9.18	8.44	8.47	8.53
IF4G1_HUMAN	0	6.4	0	0	0	12.58	0	0	11.66	0
IGF2_HUMAN	3.71	4.51	0	4.08	7.18	9.43	5.55	3.61	6.74	6.25
IGHG1_HUMAN	8.16	8.2	8.24	8.26	9.4	9.47	9.51	9.46	9.44	8.24
IGHG4_HUMAN	0	0	0	5.45	6.43	7.98	6.29	0	0	0
IGHM_HUMAN	11.44	12.32	11.48	12.48	11.25	11.42	11.92	12.08	11.54	10.04
IGKC_HUMAN	9.65	7.24	10.96	9.86	3.86	5.74	12.83	9.18	4.87	8.01
IGLL5_HUMAN	9.27	12.24	7.73	10	7.1	10.08	9.27	8.12	8.87	7.72
IHH_HUMAN	8.08	8.14	8.49	8.48	8.16	8.5	8.13	8.22	8.09	8.48
IL25_HUMAN	7	8.91	7.19	10.25	9.83	7.04	9.05	10.09	5.23	10.38
IMA8_HUMAN	0	0	0	4.23	4.6	0	0	0	4.61	7.76
IMDH1_HUMAN	6.47	6.76	7.22	8.09	4.53	6.86	7.45	6.7	5.75	6.04
IP3KB_HUMAN	5.96	4.89	3	5.83	6.81	8.14	6.53	5.06	7.09	6.14
IP3KC_HUMAN	5.95	6.84	6.54	6.93	7.47	7.38	6.54	5.68	0	7.77
IQGA1_HUMAN	2.39	5.22	3.01	5.26	3.94	4.03	4.87	4.11	4.38	7.27
IRS2_HUMAN	9.68	9.34	9.47	11.08	0	0	10.08	0	0	0
ITIH1_HUMAN	8.04	7.92	7.57	7.55	8.01	7.8	7.93	7.67	8.14	8.17
ITIH2_HUMAN	8.64	9.01	8.3	8.77	9.34	9.78	8.71	7.77	9.82	9.49
K1551_HUMAN	8.46	7.24	7.9	9.34	0	0	6.49	7.12	0	0
KCRM_HUMAN	0	0	0	0	11.51	11.45	10.2	11.51	11.47	11.44
KDM4A_HUMAN	0	9.31	9.43	0	10.37	10.63	10.05	10.37	9.34	9.32
KDM5B_HUMAN	0	1.77	6.95	4.36	0	0	7.25	0	0	0
KI20B_HUMAN	0	7.49	0	0	7.59	7.46	0	7.59	0	0
KIF27_HUMAN	6.2	5.86	5.6	6.38	6.35	8.7	7.03	7.2	6.75	8.77
KIF3B_HUMAN	6.2	5.86	5.6	6.38	6.35	8.7	7.03	7.2	6.75	8.77
KIFC2_HUMAN	13.94	14.39	13.84	0	13.97	13.98	0	13.96	13.98	13.96
KLD7B_HUMAN	13.48	13.49	13.49	13.44	14.24	0	13.47	14.24	0	0
KLD8A_HUMAN	0	9.33	0	0	0	9.42	0	0	9.64	8.96
KLH40_HUMAN	5.88	6.13	5.87	6.76	6.07	6.62	5.67	6.33	5.42	4.94
KLK6_HUMAN	0	0	0	0	10.98	11.98	0	10.98	0	0
KMT2B_HUMAN	0	0	7.57	3.67	0	0	8.92	7.63	0	0
L1CAM_HUMAN	8.41	8.31	8.36	8.37	8.31	8.32	8.3	8.32	8.33	8.37
LAC2_HUMAN	6.67	8.22	6.23	6.71	5.59	7	6.65	6.28	4.82	0
LAMA3_HUMAN	6.21	9.26	4.56	5.75	0	7.49	6.36	6.15	6.23	0
LAMB3_HUMAN	11.66	11.7	11.71	0	0	0	11.67	11.6	0	11.6
LFTY2_HUMAN	5.58	5.57	11.39	11.38	5.89	5.58	11.35	11.69	5.57	11.38
LPIN1_HUMAN	10.53	0	0	0	10.08	10.56	10.54	10.54	0	9.77
LR16A_HUMAN	13.36	13.34	0	14.71	13.34	13.36	0	13.35	13.35	14.71
LRC17_HUMAN	0	0	0	12.09	6.9	11.02	0	12.38	0	0
LRRK2_HUMAN	12.29	12.34	12.34	12.34	12.35	0	12.32	12.33	12.35	12.34
LYST_HUMAN	0	5.62	3.77	5.19	4.6	0	5.59	5.17	0	6.99
M2GD_HUMAN	13.66	13.63	13.64	13.66	0	0	13.63	13.66	0	13.66
M3K3_HUMAN	0	0	0	5.48	5.7	5.79	9.96	0	0	0
M4A10_HUMAN	0	11.71	11.68	11.75	0	0	0	0	0	11.68
MAGT1_HUMAN	8.12	7.99	8.12	8.04	8.05	8.02	7.99	8.05	8.06	8.06
MAN1_HUMAN	8.13	4.7	8.51	8.35	7.92	7.06	8.32	6.64	7.31	7.82
MAP1A_HUMAN	6.41	5.96	6.63	6.44	5.03	6.49	7.35	6.41	5.94	9.56
MAP7_HUMAN	8.54	10.41	6.65	8.03	6.14	9.35	11.07	6.54	5.74	5.24
MCA3_HUMAN	11.96	11.95	11.96	11.93	11.98	12	11.96	11.95	11.98	11.9
MCF2L_HUMAN	5.63	6.59	4.12	7.59	7.28	9.2	6.43	7.87	6.47	7.69
MED23_HUMAN	4.26	4.49	3.95	5.61	6.77	6.47	5.52	6.31	6.75	2.64
MELT_HUMAN	8.63	7.75	7.64	9.01	6.73	8.85	8.46	7.92	6.81	7.82
MFN2_HUMAN	9	9.77	7.86	10.14	0	0	8.23	8.2	0	0
MICU2_HUMAN	7.97	6.8	0	0	0	0	9.82	6.98	5.55	0
MIPO1_HUMAN	7.05	6.75	7.05	7.62	6.5	6.31	6.72	6.22	6.52	5.87
MPCP_HUMAN	6.98	8.22	0	0	10.07	8.97	8.68	0	7.11	0
MRCKA_HUMAN	0	11.04	10.16	11.02	9.95	0	11	9.97	9.99	11.02
MRP2_HUMAN	8.27	8.48	8.46	8.42	8.28	8.3	8.42	8.43	8.42	7.87
MTLR_HUMAN	10.32	10.31	10.26	10.27	10.23	10.27	10.3	10.23	10.28	10.33
MTRR_HUMAN	4.16	12.27	3.5	4.38	5.03	4.78	3.78	3.66	5.24	4.62
MUC6_HUMAN	4.37	5.62	4.65	4.29	7.59	7.38	3.84	4.2	5.92	5.62
MUCB_HUMAN	7.08	0	5.68	0	7.1	7.52	7.55	0	7.04	9.08
MUS81_HUMAN	6.21	6.62	6.1	5.46	5.66	6.64	5.87	6.37	6.12	7.31
MYCB2_HUMAN	6.47	5.9	5.68	6.31	7.38	4.62	6.79	5.99	7.63	7.45
MYCT_HUMAN	10.28	9.05	10.26	9.1	10.17	10.25	10.31	10.19	10.22	10.26
MYL10_HUMAN	5.47	0	8.14	8.07	5.81	0	13.08	7.92	5.83	5.86
MYPN_HUMAN	8.42	8.23	8.34	9.84	7.97	8.42	8.4	9.9	8.49	8.84
MYT1L_HUMAN	4.23	5.29	0	3.61	4.8	5.26	5.38	5.07	4.9	9.69
NAR4_HUMAN	8.56	8.74	10.78	10.76	10.39	8.69	10.7	8.67	10.73	10.68
NBEA_HUMAN	9.3	8	9.33	8.56	8.97	8.05	8.06	8.97	9.33	10.05

The 392 proteins with MS identified and Uniprot ID from 6 patients (P) and 4 donors (D)

Uniprot KB	P1	P2	P3	P4	P5	P6	D1	D2	D3	D4
NCYM_HUMAN	9.6	9.62	9.31	0	9.66	9.66	9.6	9.59	9.64	9.59
NEBL_HUMAN	4.39	4.44	4.57	4.79	6.52	6.88	6.02	5.45	6.37	5.76
NEDD1_HUMAN	8.35	9	8.38	8.99	0	0	8.5	0	8.36	8.99
NEUG_HUMAN	7.85	0	7.47	5.95	8.03	4.97	9.65	8.11	7.12	7.22
NICA_HUMAN	11.56	11.59	11.58	11.59	11.75	0	11.58	11.75	0	0
NIN_HUMAN	7.23	0	0	7.62	0	4.37	8.58	0	0	0
NLRC3_HUMAN	0	0	5.84	5.81	5.96	7.85	6.17	5.85	0	5.94
NMNA1_HUMAN	9.85	10.4	10.67	10.32	9.82	0	9.74	10.7	10.58	10.32
NRX1A_HUMAN	13.06	11.59	13.1	11.57	13.07	0	13.1	13.07	0	0
NUCB1_HUMAN	10.68	11.14	10.68	11.13	10.7	0	10.66	10.69	10.64	11.13
NXF1_HUMAN	8.39	8.48	8.45	8.48	8.44	8.44	8.46	8.46	8.43	8.48
OBSCN_HUMAN	8.66	8.58	8.66	8.66	8.56	8.56	8.62	8.56	8.61	8.6
ODF3A_HUMAN	0	0	0	0	3.48	3.87	0	0	0	5.83
OR1M1_HUMAN	12.45	12.46	12.45	12.48	12.5	0	12.47	12.48	12.48	12.48
OR9Q2_HUMAN	10.38	10.41	0	0	10.43	10.45	10.39	10.34	10.39	10.34
P3C2B_HUMAN	10.78	10.79	10.78	10.79	11.75	11.76	10.78	11.71	11.76	11.71
P85B_HUMAN	10.3	10.08	10.27	10.24	11.1	10.54	10.16	11.1	10.53	10.54
PABP5_HUMAN	10.25	10.25	10.26	10.29	10.19	11.56	10.27	10.19	10.22	10.26
PALMD_HUMAN	0	0	12.58	7.73	0	0	11.91	11.43	11.69	0
PARP8_HUMAN	0	0	10.24	0	9.65	0	10.21	9.65	0	10.25
PB1_HUMAN	0	3.56	2.99	5.07	5.6	0	0	4.39	4.86	9.17
PC11X_HUMAN	5.02	0	0	0	7.07	6.1	0	0	5.1	5.94
PCKGM_HUMAN	0	12.94	12.93	12.95	0	0	0	0	0	12.93
PCLO_HUMAN	11.31	0	11.34	11.32	11.73	11.34	11.3	11.73	11.32	11.3
PDE11_HUMAN	5.96	7.29	5.34	2.38	0	2.88	5.98	6.41	6.63	8.73
PDE4D_HUMAN	9.93	9.89	6.85	9.81	10.32	10.3	9.73	10.17	10.45	10.17
PDE6D_HUMAN	6.36	6.11	5.7	6.26	7.56	3.45	6.91	5.94	7.87	7.12
PDS5B_HUMAN	10.32	10.39	10.39	10.39	10.46	10.45	10.34	10.38	10.43	10.39
PE2R2_HUMAN	7.22	8.66	8.75	8.05	5.11	5.05	9.38	5.91	6.2	5.98
PEX1_HUMAN	5.96	5.68	6.13	8.47	4.52	5.64	5.77	6.82	3.94	5.05
PGM2_HUMAN	13.35	13.36	13.36	13.89	0	0	13.34	14.53	0	14.53
PICK1_HUMAN	0	9.2	0	0	0	9.41	8.73	0	0	8.05
PIWL1_HUMAN	7.26	5.84	6.67	7.79	3.82	5.35	0	6.31	5.22	5.14
PKHA1_HUMAN	0	0	0	0	0	10.96	0	10.92	10.95	10.92
PKP3_HUMAN	7	5.92	6.82	9.13	5.9	3.98	8.99	7.8	6.73	10.04
PLAG1_HUMAN	5.27	5.6	0	0	6.92	6.42	4.43	0	5.13	0
PLCD3_HUMAN	10.51	10.53	10.5	10.5	10.55	10.54	10.5	10.53	10.53	10.5
PLMN_HUMAN	5.86	5.78	4.38	7.47	7.41	4.5	5.16	5.87	3.8	0
PLXA4_HUMAN	0	10.48	0	0	10.5	10.53	10.46	10.47	10.52	10.47
PLXD1_HUMAN	0	0	0	4.86	6.84	8.96	3.21	0	6.84	5.66
PPLA_HUMAN	12.67	12.69	12.69	12.7	12.71	0	12.7	12.71	12.73	12.7
PPRC1_HUMAN	10.43	10.46	10.39	10.43	10.35	11.36	10.45	10.37	10.37	10.39
PRDM9_HUMAN	7.78	0	7.86	7.86	7.81	7.76	7.83	0	7.89	7.86
PRKDC_HUMAN	12.26	10.36	0	0	12.29	12.29	10.62	12.28	12.29	12.25
PRP19_HUMAN	4.72	5.68	3.57	3.66	7.6	5.98	3.86	4.34	5.84	5.56
PSA4_HUMAN	8.38	8.4	9.35	0	0	9.31	9.29	0	0	9.1
PTPRT_HUMAN	6.38	5.49	5.54	6.13	6.54	7.53	6.39	5.73	7.36	7.09
RA1L2_HUMAN	0	7.12	0	0	0	7.01	6.04	6.73	0	8.6
RADIL_HUMAN	6.8	7.07	6.28	6.09	7.82	7.68	6.17	6.78	7.33	7.3
RAG2_HUMAN	12.27	12.3	11.68	10.82	12.23	0	12.3	0	0	11.68
RBM5_HUMAN	7.06	6.18	6.78	6.93	0	4.33	7.03	6.37	5.41	5.79
RBMX_HUMAN	4.24	5.62	1.74	0	8.72	8.2	3.27	2.89	6.67	0
RBPJL_HUMAN	4.29	5.5	2.32	1.57	8.78	8.32	3.4	2.8	6.76	6.05
RBY1F_HUMAN	4.93	7.51	0	5.17	5.37	6.74	5.69	5.79	5.52	8.52
RCAS1_HUMAN	4.57	6.04	2.61	2.75	8.12	8.1	3.74	3.49	6.59	5.83
RCBT2_HUMAN	13.86	13.86	13.86	13.83	13.86	0	13.87	13.86	0	0
RENT2_HUMAN	7.59	6.91	6	6.18	7.18	9.04	7.27	7.07	8.73	8.92
REPI1_HUMAN	9.21	9.86	9.18	8.41	9.81	0	8.2	9.84	9.2	8.41
RERGL_HUMAN	9.87	0	0	10.37	4.92	3.59	4.39	0	2.04	5.04
RFX1_HUMAN	9.45	9.45	9.45	9.41	9.45	9.4	9.4	9.45	9.42	9.41
RGS3_HUMAN	6.58	4.94	6	6.37	5.64	6.55	6.22	5.7	6.1	7.35
RHF2B_HUMAN	0	0	7.57	3.67	0	0	8.92	7.63	0	0
RHG22_HUMAN	2.06	6.32	0	1.44	4.53	6.12	2.15	1.72	4.4	8.71
RHXF2_HUMAN	0	0	7.57	3.67	0	0	8.92	7.63	0	0
RICTR_HUMAN	11.62	11.63	11.67	11.66	12.08	0	11.64	12.08	0	0
RIMS1_HUMAN	11.55	0	11.58	0	11.76	0	11.57	11.76	0	0
RIPK4_HUMAN	4.64	6.33	6.92	9.25	6.73	5.93	6.93	5.81	6.77	7.48
RIR2B_HUMAN	5.21	0	5	0	0	7.3	7.96	5.86	0	9.78
RL13_HUMAN	3.69	0	0	4.34	4.43	9.5	4.5	0	3.01	6.8
RM17_HUMAN	13.26	13.26	13.27	13.25	0	0	13.29	13.28	0	13.28
ROP1L_HUMAN	2.79	6.21	0	8.26	2.31	0	2.74	6.42	0	0
RPOM_HUMAN	10.77	10.73	10.78	10.68	10.79	0	0	0	0	10.68
RPTOR_HUMAN	5.91	6.08	0	9.38	0	6.58	9.91	5.55	0	4.02
RREB1_HUMAN	9.41	7.12	7.97	7.35	10.79	12.04	8.38	9.79	8.4	10.73
RTL1_HUMAN	9.43	9.25	9.44	9.33	9.33	9.32	9.39	9.33	9.33	9.35
S14L2_HUMAN	8.97	9.47	0	8.35	8.53	8.51	8.83	8.42	8.31	8.42

The 392 proteins with MS identified and Uniprot ID from 6 patients (P) and 4 donors (D)

Uniprot KB	P1	P2	P3	P4	P5	P6	D1	D2	D3	D4
S1PR3_HUMAN	8.35	8.27	8.36	8.34	8.19	8.27	8.29	8.19	8.28	8.32
S2610_HUMAN	4.53	5.59	5.58	8.7	0	0	6.24	0	0	0
S31E1_HUMAN	4.09	4.68	0	2.3	7.63	7.01	3.31	5.41	3.56	0
S38A4_HUMAN	9.67	9.65	9.62	9.65	8.21	9.59	9.52	8.21	9.63	8.05
S40A1_HUMAN	3.6	4.79	4.52	5.71	8.08	5.67	5.91	6.15	6.28	5.8
SAC31_HUMAN	0	3.12	5.39	7.75	2.71	4.1	4.53	0	5.6	5.47
SACS_HUMAN	9.74	10.02	9.93	9.3	9.25	9.93	9.91	0	9.98	9.3
SCAPE_HUMAN	7.24	9.63	6.54	10.49	2.56	5.65	8.32	7.88	3.8	3.93
SCN3A_HUMAN	11.37	11.38	11.36	11.36	11.29	0	11.33	11.39	11.3	11.36
SETD2_HUMAN	11.75	10.8	11.73	11.7	11.76	0	10.8	12.35	11.75	11.7
SETD5_HUMAN	7.66	7.39	6.26	9.67	0	0	8.27	6.25	0	0
SGPL1_HUMAN	0	7.34	0	0	6.22	6.75	6.74	6.66	7.18	7.59
SH3K1_HUMAN	7.53	7.29	4.7	4.9	4.73	4.17	5.11	4.48	6.04	5.85
SHAN3_HUMAN	9.57	9.7	9.47	8.59	11.78	11.33	10.42	9.68	9.63	10.54
SHH_HUMAN	13.15	13.09	13.15	13.17	0	0	13.15	13.19	0	13.19
SKDA1_HUMAN	11.45	11.46	11.47	12.69	0	11.5	11.46	12.14	11.47	12.14
SL9A5_HUMAN	0	0	0	12	0	0	9.98	0	12	12
SMC2_HUMAN	9.82	9.83	9.79	9.85	9.79	10.9	9.84	9.72	9.76	9.79
SMYD2_HUMAN	4.59	4.34	4.19	7.83	0	4.48	4.52	0	5.92	0
SNPC4_HUMAN	12.73	12.74	12.77	13.1	0	0	12.76	13.13	0	13.13
SNTB2_HUMAN	0	0	5.65	2.94	0	0	8.07	7.65	0	0
SO2A1_HUMAN	4.08	4.52	0	0	9.58	6.06	0	0	4.36	5.46
SOS1_HUMAN	5.83	4.64	3.17	2.95	8.07	6.95	3.87	4.53	4.21	5.15
SPAT7_HUMAN	6.01	4.62	0	5.62	5.34	6.62	0	0	4.6	6.12
SPEG_HUMAN	8.13	7.29	12.61	12.4	5.74	0	7.99	0	8.94	4.91
SPG7_HUMAN	7.73	7.85	7.84	7.85	7.77	7.74	7.81	7.87	7.85	7.85
SPRY3_HUMAN	0	12.23	12.23	12.21	12.22	0	12.2	12.2	12.22	12.21
SPTN4_HUMAN	0	0	0	0	9.55	10.41	0	9.55	10.42	9.72
STK16_HUMAN	5.68	6.31	6.1	9.43	5.67	9.8	5.66	10.23	9.94	10.83
SUZ12_HUMAN	9.56	9.57	9.58	9.56	9.56	9.55	9.51	9.59	9.57	9.56
SYCP3_HUMAN	4.57	5.08	4.53	4.16	7.63	7.27	3.51	4.34	6.09	5.51
SYTL2_HUMAN	4.5	5.5	0	4.33	3.91	4.07	3.44	3.13	3.35	2.88
SYYM_HUMAN	8.04	8.07	8.02	8.01	7.96	7.93	7.95	7.96	8	8.01
T11L1_HUMAN	7.93	7.86	6.91	8.56	0	0	6.76	6.83	0	0
TANC2_HUMAN	13.99	13.98	13.98	13.96	12.52	0	13.99	12.52	0	0
TB10B_HUMAN	10.55	10.59	10.57	0	10.63	10.57	10.55	10.63	10.52	10.56
TBD2A_HUMAN	6.96	6.32	6.54	7.28	0	0	6.24	6.23	0	0
TCPB_HUMAN	9.25	9.33	8.09	10.81	0	0	9.44	7.62	0	0
TEN3_HUMAN	10.33	10.39	0	0	10.37	10.41	10.33	10.4	10.42	10.38
TEX2_HUMAN	8.34	8.41	8.36	8.43	8.23	8.35	0	8.3	8.39	8.36
TF2H4_HUMAN	13.59	13.19	13.21	13.18	13.07	0	13.19	13.07	0	0
TGT_HUMAN	8.22	7.67	8.24	7.59	0	0	8.2	8.13	0	0
THRB_HUMAN	5.94	6.5	5.52	7.45	6.39	5.65	6.91	7.12	7.36	7.78
TIGD1_HUMAN	4.29	6.42	4.56	5.95	7.56	9.34	7.98	7.25	11.51	12.09
TIMP4_HUMAN	3.3	4.97	0	4.91	5.84	6.94	4.91	4.53	6.09	7.11
TITIN_HUMAN	4.74	0	0	6.51	9.12	10.96	6.18	0	0	0
TM119_HUMAN	4.88	7.34	6.21	6.59	4.21	6.92	5.9	5.08	6.51	5.35
TM175_HUMAN	5.9	6.37	6.86	8.95	5.95	3.5	7.99	3.93	5.83	0
TM40L_HUMAN	5.99	6.87	6.28	6.29	0	0	6.49	5.65	0	0
TMED5_HUMAN	10.08	10.04	10.07	10.07	11.19	10.85	10.05	11.19	10.38	11.18
TMM78_HUMAN	9.42	8.84	8.79	8.73	10.2	10.24	9.43	9.49	9.34	8.79
TPIS_HUMAN	6.34	6.47	4.95	8.96	0	0	7.47	5.2	0	0
TRFE_HUMAN	5.21	0	1.69	3.29	4.42	6.89	5.52	6.21	7.09	8.22
TRIM3_HUMAN	10.48	10.44	10.45	10.38	10.45	0	10.44	10.48	10.47	10.38
TRPS1_HUMAN	13.67	13.64	13.64	13.67	0	0	13.64	13.69	0	13.69
TRY1_HUMAN	10.35	10.42	10.37	10.38	10.43	10.42	10.39	10.41	10.39	10.38
TSC2_HUMAN	3.89	6.93	0	2.75	0	3.21	2.89	1.77	0	0
TT39C_HUMAN	4.72	3.92	3.33	5.14	6.98	7.73	5.53	3.99	6.98	5.89
TTHY_HUMAN	10.25	10.26	10.26	10.28	10.28	10.27	10.25	10.24	10.26	10.24
TVB2_HUMAN	5.27	4.94	0	5.12	8.3	8.11	4.63	4.42	3.23	4.66
U3IP2_HUMAN	13.07	13.08	13.1	13.08	13.08	0	13.09	13.08	0	0
UB2V2_HUMAN	10.45	10.47	9.57	9.66	10.31	0	9.3	9.56	9.57	9.57
UBP2L_HUMAN	8.44	0	8.42	8.35	8.34	8.34	8.29	8.34	8.36	8.4
VAS1_HUMAN	13.74	13.73	13.74	13.69	13.05	0	13.75	13.05	0	0
VASH1_HUMAN	5.94	0	0	6.93	6.58	9.02	7.65	0	0	0
VAV3_HUMAN	0	4.8	0	5.16	0	4.89	0	5.61	5.7	8.32
VGFR2_HUMAN	6.85	6.87	6.52	6.27	6.31	6.16	7.07	6.37	6.44	5.97
VGFR3_HUMAN	7.46	4.76	8.24	3.22	0	5.49	0	5.37	0	0
VITRN_HUMAN	0	7.22	5.19	3.37	5.52	5.97	10.28	10.89	5.01	4.99
VPS11_HUMAN	6.16	6.77	6.13	5.66	5.85	6.9	6.01	6.43	6.71	4.82
WDR12_HUMAN	5.92	6.48	6.03	5.14	5.43	6.55	5.59	5.97	5.81	7.63
WWP1_HUMAN	11.7	11.68	11.63	11.7	11.68	0	11.68	11.71	11.72	11.7
XKR7_HUMAN	8.23	7.82	7.77	7.43	6.61	7.6	8.55	8.44	7.38	7.71
XXLT1_HUMAN	11.17	11.21	11.18	11.16	11.16	11.17	11.2	11.17	11.2	11.16
Z3H7B_HUMAN	5.23	5.52	0	5.01	6.34	5.93	0	0	4.05	7.75
ZBBX_HUMAN	9.85	9.84	9.82	9.56	4.76	5.21	9.87	4.44	4.46	9.82

The 392 proteins with MS identified and Uniprot ID from 6 patients (P) and 4 donors (D)

Uniprot KB	P1	P2	P3	P4	P5	P6	D1	D2	D3	D4
ZC12A_HUMAN	4.36	0	0	5.79	5.6	7.97	5.77	0	0	0
ZC3H1_HUMAN	13.64	0	12.68	12.4	0	0	12.39	12.09	0	12.09
ZMY15_HUMAN	8.58	8.63	8.36	8.61	8.56	8.46	8.49	8.61	8.42	8.58
ZN202_HUMAN	6.52	6.2	5.84	6.37	7.51	8.38	6.79	6.11	7.97	7.67
ZN250_HUMAN	0	0	0	0	11.04	0	0	11.04	0	11.05
ZN471_HUMAN	0	2.98	0	4.24	6.6	7.92	4.7	3.29	7.41	5.57
ZN609_HUMAN	8.24	9.99	0	0	8.24	8.16	9.93	0	8.41	9.93
ZN815_HUMAN	0	5.07	0	4.59	0	5.81	5.89	0	5.41	9.65
ZNT10_HUMAN	4.53	5.21	5.51	5.15	7.04	6.57	3.16	6.14	7.24	7.85
ZRAB3_HUMAN	13.75	13.73	13.73	13.74	0	0	13.75	13.79	0	13.79
ZSC30_HUMAN	5.71	6.47	5.89	5.3	5.44	6.49	5.63	5.93	5.86	7.59
ZSCA1_HUMAN	8.53	8.52	8.49	8.5	8.43	8.43	8.44	8.42	8.47	8.5

Table S2

The 106 secretory proteins with biological process chracterization

Uniprot KB	Protein name	Gene	Biological process (www.uniprot.org)
A2MG_HUMAN	Alpha-2-macroglobulin	A2M	blood coagulation, intrinsic pathway<br>extracellular matrix disassembly<br>negative regulation of complement activation, lectin pathway platelet degranulation<br>regulation of small GTPase mediated signal -transduction<br>stem cell differentiation
ABCB6_HUMAN	ATP-binding cassette sub-family B member 6, mitochondrial	ABCB6	brain development<br>cellular iron ion homeostasis<br>heme transport<br>porphyrin-containing compound biosynthetic process<br>skin development<br>transmembrane transport<br>transport
BGAT_HUMAN	Histo-blood group ABO system transferase	ABO	carbohydrate metabolic process<br>glycolipid biosynthetic process<br>lipid glycosylation<br>protein glycosylation
ACLY_HUMAN	ATP-citrate synthase	ACLY	acetyl-CoA biosynthetic process<br>cholesterol biosynthetic process<br>citrate metabolic process<br>fatty acid biosynthetic process<br>lipid biosynthetic process<br>long-chain fatty-acyl-CoA biosynthetic process<br>neutrophil degranulation<br>oxaloacetate metabolic process<br>positive regulation of cellular metabolic process

AL7A1_HUMAN	Alpha-aminoadipic semialdehyde dehydrogenase	ALDH7A1	cellular aldehyde metabolic process<br>choline catabolic process<br>glycine betaine biosynthetic process from choline<br>lysine catabolic process<br>sensory perception of sound
ANGL7_HUMAN	Angiopoietin-related protein 7	ANGPTL7	response to oxidative stress

APOA1_HUMAN	Apolipoprotein A-I	APOA1	endothelial cell proliferation<br>glucocorticoid metabolic process<br>G-protein coupled receptor signaling pathway<br>high-density lipoprotein particle assembly<br>high-density lipoprotein particle clearance<br>high-density lipoprotein particle remodeling<br>integrin-mediated signaling pathway<br>negative regulation of cell adhesion molecule production<br>negative regulation of cytokine secretion involved in immune response<br>negative regulation of heterotypic cell-cell adhesion<br>negative regulation of inflammatory response<br>negative regulation of interleukin-1 beta secretion<br>negative regulation of lipase<br>negative regulation of response to cytokine stimulus<br>negative regulation of tumor necrosis factor-mediated signaling pathway negative<br>regulation of very-low-density lipoprotein particle remodeling<br>platelet degranulation<br>positive regulation of cholesterol esterification<br>positive regulation of fatty acid biosynthetic process<br>positive regulation of hydrolase activity<br>positive regulation of lipoprotein lipase activity<br>positive regulation of Rho protein signal transduction<br>positive regulation of stress fiber assembly<br>positive regulation of substrate adhesion-dependent cell spreading<br>positive regulation of triglyceride catabolic process<br>protein oxidation<br>protein stabilization<br>receptor-mediated endocytosis<br>regulation of Cdc42 protein signal transduction<br>regulation of intestinal cholesterol absorption<br>regulation of protein phosphorylation<br>[illegible]

APOA2_HUMAN	Apolipoprotein A-II	APOA2	negative regulation of cholesterol import<br>negative regulation of cholesterol transport<br>negative regulation of cholesterol transporter activity<br>negative regulation of cytokine secretion involved in immune response<br>negative regulation of lipase activity<br>negative regulation of lipid catabolic process<br>negative regulation of very-low-density lipoprotein particle remodeling<br>positive regulation of cholesterol esterification Source: BHF-UCL<br>positive regulation of interleukin-8 biosynthetic process Source: UniProtKB<br>positive regulation of lipid catabolic process Source: BHF-UCL<br>protein oxidation
APOD_HUMAN	Apolipoprotein D	APOD	aging<br>angiogenesis<br>glucose metabolic process<br>lipid metabolic process<br>negative regulation of cytokine production involved in inflammatory response<br>negative regulation of focal adhesion assembly<br>negative regulation of lipoprotein lipid oxidation<br>negative regulation of monocyte chemotactic protein-1 production<br>negative regulation of platelet-derived growth factor receptor signaling pathway<br>negative regulation of protein import into nucleus<br>negative regulation of smooth muscle cell-matrix adhesion<br>negative regulation of smooth muscle cell proliferation<br>negative regulation of T cell migration<br>response to reactive oxygen species<br>tissue regeneration
VAS1_HUMAN	V-type proton ATPase subunit S1	ATP6AP1	ATP hydrolysis coupled proton transport<br>establishment of organelle localization<br>insulin receptor signaling pathway<br>ion transmembrane transport<br>positive regulation of bone resorption<br>positive regulation of ERK1 and ERK2 cascade<br>positive regulation of exocytosis<br>positive regulation of osteoblast differentiation<br>positive regulation of osteoclast development

BCAM_HUMAN	Basal cell adhesion molecule	BCAM	cell adhesion<br>cell-matrix adhesion<br>signal transduction
CU025_HUMAN	C2 domain-containing protein 2	C2CD2	
CO3_HUMAN	Complement C3	C3	complement activation<br>complement activation, alternative pathway<br>complement activation, classical pathway<br>fatty acid metabolic process<br>G-protein coupled receptor signaling pathway<br>immune response<br>inflammatory response<br>neutrophil degranulation<br>positive regulation of activation of membrane attack complex<br>positive regulation of angiogenesis<br>positive regulation of apoptotic cell clearance<br>positive regulation of glucose transport<br>positive regulation of G-protein coupled receptor protein signaling pathway<br>positive regulation of lipid storage<br>positive regulation of protein phosphorylation<br>positive regulation of type IIa hypersensitivity<br>positive regulation of vascular endothelial growth factor production<br>regulation of complement activation<br>regulation of immune response<br>regulation of triglyceride biosynthetic process<br>signal transduction
CO4A_HUMAN	Complement C4-A	C4A	complement activation<br>complement activation, classical pathway<br>inflammatory response<br>innate immune response<br>positive regulation of apoptotic cell clearance<br>regulation of complement activation
CAH6_HUMAN	Carbonic anhydrase 6	CA6	

CAN7_HUMAN	Calpain-7	CAPN7	positive regulation of epithelial cell migration<br>self proteolysis
CAZA1_HUMAN	F-actin-capping protein subunit alpha-1	CAPZA1	barbed-end actin filament capping<br>blood coagulation<br>innate immune response<br>movement of cell or subcellular component<br>protein complex assembly
TCPB_HUMAN	T-complex protein 1 subunit beta	CCT2	chaperone-mediated protein complex assembly<br>chaperone mediated protein folding independent of cofactor<br>neutrophil degranulation<br>positive regulation of establishment of protein localization to telomere<br>positive regulation of protein localization to Cajal body<br>positive regulation of telomerase activity<br>positive regulation of telomerase RNA localization to Cajal body<br>positive regulation of telomere maintenance via telomerase<br>protein folding
MRCKA_HUMAN	Serine/threonine-protein kinase MRCK alpha	CDC42BPA	actin cytoskeleton reorganization<br>actomyosin structure organization<br>cell migration<br>intracellular signal transduction<br>protein phosphorylation
CK5P2_HUMAN	CDK5 regulatory subunit-associated protein 2	CDK5RAP2	brain development<br>centriole replication<br>centrosome organization<br>chromosome segregation<br>ciliary basal body docking<br>establishment of mitotic spindle orientation<br>G2/M transition of mitotic cell cycle<br>microtubule bundle formation<br>microtubule cytoskeleton organization<br>microtubule organizing center organization<br>negative regulation of centriole replication<br>negative regulation of neuron differentiation<br>neurogenesis<br>positive regulation of transcription, DNA-templated<br>regulation of neuron differentiation<br>regulation of spindle checkpoint

CP250_HUMAN	Centrosome-associated protein CEP250	CEP250	centriole-centriole cohesion<br>ciliary basal body docking<br>G2/M transition of mitotic cell cycle<br>mitotic cell cycle<br>non-motile cilium assembly<br>positive regulation of protein localization to centrosome<br>protein localization<br>protein localization to organelle<br>regulation of centriole-centriole cohesion

CLUS_HUMAN	Clusterin	CLU	cell morphogenesis<br>central nervous system myelin maintenance<br>chaperone-mediated protein complex assembly<br>chaperone-mediated protein folding<br>complement activation<br>complement activation, classical pathway<br>innate immune response<br>intrinsic apoptotic signaling pathway<br>lipid metabolic process<br>microglial cell activation<br>microglial cell proliferation<br>negative regulation of amyloid fibril formation<br>negative regulation of beta-amyloid formation<br>negative regulation of cell death<br>negative regulation of cellular response to thapsigargin<br>negative regulation of cellular response to tunicamycin<br>negative regulation of intrinsic apoptotic signaling pathway in response to DNA damage<br>negative regulation of protein homooligomerization<br>negative regulation of release of cytochrome c from mitochondria<br>negative regulation of response to endoplasmic reticulum stress<br>platelet degranulation<br>positive regulation of apoptotic process<br>positive regulation of beta-amyloid formation<br>positive regulation of intrinsic apoptotic signaling pathway<br>positive regulation of neurofibrillary tangle assembly<br>positive regulation of neuron death<br>positive regulation of NF-kappaB transcription factor activity<br>positive regulation of nitric oxide biosynthetic process<br>positive regulation of proteasomal ubiquitin-dependent protein catabolic process<br>positive regulation of protein homooligomerization<br>[illegible]

CO2A1_HUMAN	Collagen alpha-1(II) chain	COL2A1	cartilage condensation<br>cartilage development<br>cartilage development involved in endochondral bone morphogenesis<br>cellular response to BMP stimulus<br>central nervous system development<br>chondrocyte differentiation<br>collagen catabolic process<br>collagen fibril organization<br>embryonic skeletal joint morphogenesis<br>endochondral ossification<br>extracellular matrix organization<br>heart morphogenesis<br>negative regulation of extrinsic apoptotic signaling pathway in absence of ligand<br>palate development<br>proteoglycan metabolic process<br>regulation of gene expression<br>regulation of immune response<br>skeletal system development<br>tissue homeostasis
CO6A3_HUMAN	Collagen alpha-3(VI) chain	COL6A3	cell adhesion<br>collagen catabolic process<br>extracellular matrix organization<br>muscle organ development
CERU_HUMAN	Ceruloplasmin	CP	cellular iron ion homeostasis<br>copper ion transport
CBPD_HUMAN	Carboxypeptidase D	CPD	peptide metabolic process<br>protein processing

CTNB1_HUMAN	Catenin beta-1	CTNNB1	adherens junction assembly<br>androgen receptor signaling pathway<br>anterior/posterior axis specification<br>beta-catenin destruction complex disassembly<br>beta-catenin-TCF complex assembly<br>bone resorption<br>branching involved in blood vessel morphogenesis<br>branching involved in ureteric bud morphogenesis<br>canonical Wnt signaling pathway<br>canonical Wnt signaling pathway involved in midbrain dopaminergic neuron differentiation<br>canonical Wnt signaling pathway involved in negative regulation of apoptotic process<br>canonical Wnt signaling pathway involved in positive regulation of cardiac outflow tract cell proliferation<br>canonical Wnt signaling pathway involved in positive regulation of epithelial to mesenchymal transition<br>catenin import into nucleus<br>cell adhesion<br>cell fate specification<br>cell-matrix adhesion<br>cell maturation<br>cell morphogenesis involved in differentiation<br>cellular response to growth factor stimulus<br>endodermal cell fate commitment<br>endothelial tube morphogenesis<br>epithelial cell differentiation involved in prostate gland development<br>epithelial to mesenchymal transition genitalia morphogenesis<br>mesenchymal cell proliferation involved in lung development<br>negative regulation of apoptotic signaling pathway<br>negative regulation of cell proliferation<br>negative regulation of chondrocyte differentiation<br>[illegible]

CATL1_HUMAN	Cathepsin L1	CTSL	adaptive immune response<br>antigen processing and presentation<br>antigen processing and presentation of exogenous peptide antigen via MHC class II<br>cellular response to thyroid hormone stimulus<br>collagen catabolic process<br>extracellular matrix disassembly<br>macrophage apoptotic process<br>proteolysis involved in cellular protein catabolic process<br>toll-like receptor signaling pathway

CUL3_HUMAN	Cullin-3	CUL3	anaphase-promoting complex-dependent catabolic process<br>cell cycle arrest<br>cell migration<br>cell projection organization<br>COPII vesicle coating<br>ER to Golgi vesicle-mediated transport<br>fibroblast apoptotic process<br>G1/S transition of mitotic cell cycle<br>gastrulation integrin-mediated signaling pathway<br>intrinsic apoptotic signaling pathway<br>MAPK cascade<br>mitotic metaphase plate congression<br>negative regulation of canonical Wnt signaling pathway<br>negative regulation of Rho protein signal transduction<br>negative regulation of transcription from RNA polymerase II promoter<br>positive regulation of cell proliferation<br>positive regulation of cytokinesis<br>positive regulation of mitotic metaphase/anaphase transition<br>proteasome-mediated ubiquitin-dependent protein catabolic process<br>protein monoubiquitination<br>protein polyubiquitination<br>protein ubiquitination<br>protein ubiquitination involved in ubiquitin-dependent protein catabolic process<br>stem cell division<br>stress fiber assembly<br>ubiquitin-dependent catabolism of misfolded proteins by nucleus-associated proteasome<br>ubiquitin-dependent protein catabolic process<br>Wnt signaling pathway

CUL4B_HUMAN	Cullin-4B	CUL4B	cell cycle<br>DNA damage response, detection of DNA damage<br>global genome nucleotide-excision repair<br>histone H2A monoubiquitination<br>neuron projection development<br>nucleotide-excision repair, DNA damage recognition<br>nucleotide-excision repair, DNA duplex unwinding<br>nucleotide-excision repair, DNA incision<br>nucleotide-excision repair, DNA incision, 3'-to lesion<br>nucleotide-excision repair, DNA incision, 5'-to lesion<br>nucleotide-excision repair, preincision complex assembly<br>nucleotide-excision repair, preincision complex stabilization<br>positive regulation of G1/S transition of mitotic cell cycle<br>positive regulation of protein catabolic process<br>protein ubiquitination involved in ubiquitin-dependent protein catabolic process<br>transcription-coupled nucleotide-excision repair
CUX2_HUMAN	Homeobox protein cut-like 2	CUX2	cellular response to organic substance<br>Golgi vesicle transport<br>multicellular organism development<br>negative regulation of transcription from RNA polymerase II promoter<br>positive regulation of dendrite morphogenesis<br>positive regulation of dendritic spine morphogenesis<br>positive regulation of excitatory postsynaptic potential<br>positive regulation of gene expression<br>positive regulation of synapse assembly<br>short-term memory<br>transcription, DNA-templated
CYB5_HUMAN	Cytochrome b5	CYB5A	L-ascorbic acid metabolic process<br>response to cadmium ion

DDX5_HUMAN	Probable ATP-dependent RNA helicase DDX5	DDX5	alternative mRNA splicing, via spliceosome<br>androgen receptor signaling pathway<br>cell growth<br>intracellular estrogen receptor signaling pathway<br>intrinsic apoptotic signaling pathway by p53 class mediator<br>mRNA splicing, via spliceosome<br>mRNA transcription<br>negative regulation of transcription from RNA polymerase II promoter<br>nuclear-transcribed mRNA catabolic process<br>positive regulation of DNA damage response, signal transduction by p53 class mediator<br>positive regulation of intracellular estrogen receptor signaling pathway<br>positive regulation of transcription from RNA polymerase II promoter<br>regulation of alternative mRNA splicing, via spliceosome<br>regulation of androgen receptor signaling pathway<br>regulation of osteoblast differentiation<br>regulation of transcription from RNA polymerase II promoter<br>RNA secondary structure unwinding
DHH_HUMAN	Desert hedgehog protein	DHH	cell-cell signaling<br>Leydig cell differentiation<br>male sex determination<br>myelination<br>osteoblast differentiation<br>regulation of steroid biosynthetic process<br>response to estradiol<br>response to estrogen<br>smoothened signaling pathway<br>spermatid development

DYST_HUMAN	Dystonin	DST	cell adhesion<br>cell motility<br>cytoskeleton organization<br>hemidesmosome assembly<br>integrin-mediated signaling pathway<br>intermediate filament cytoskeleton organization<br>maintenance of cell polarity<br>microtubule cytoskeleton organization<br>response to wounding<br>retrograde axonal transport
DYHC2_HUMAN	Cytoplasmic dynein 2 heavy chain 1	DYNC2H1	asymmetric protein localization<br>coronary vasculature development<br>determination of left/right symmetry<br>dorsal/ventral pattern formation<br>embryonic limb morphogenesis<br>forebrain development<br>Golgi organization<br>intraciliary retrograde transport<br>intraciliary transport involved in cilium assembly<br>non-motile cilium assembly<br>positive regulation of smoothened signaling pathway<br>protein processing<br>spinal cord motor neuron differentiation
MCA3_HUMAN	Eukaryotic translation elongation factor 1 epsilon-1	EEF1E1	negative regulation of cell proliferation<br>positive regulation of apoptotic process<br>positive regulation of cellular senescence<br>positive regulation of DNA damage response, signal transduction by p53 class mediator<br>tRNA aminoacylation for protein translation
FBLN3_HUMAN	EGF-containing fibulin-like extracellular matrix protein 1	EFEMP1	camera-type eye development<br>embryonic eye morphogenesis<br>epidermal growth factor receptor signaling pathway<br>negative regulation of chondrocyte differentiation<br>peptidyl-tyrosine phosphorylation<br>post-embryonic eye morphogenesis<br>regulation of transcription, DNA-templated

THRB_HUMAN	Prothrombin, Coagulation Factor II (Thrombin)	F2	acute-phase response<br>blood coagulation<br>blood coagulation, intrinsic pathway<br>cell surface receptor signaling pathway<br>cellular protein metabolic process<br>ER to Golgi vesicle-mediated transport<br>fibrinolysis<br>leukocyte migration<br>multicellular organism development<br>negative regulation of astrocyte differentiation<br>negative regulation of fibrinolysis<br>negative regulation of platelet activation<br>negative regulation of proteolysis<br>peptidyl-glutamic acid carboxylation<br>platelet activation<br>positive regulation of blood coagulation<br>positive regulation of cell growth<br>positive regulation of cell proliferation<br>positive regulation of collagen biosynthetic process<br>positive regulation of lipid kinase activity<br>positive regulation of phosphatidylinositol 3-kinase signaling<br>positive regulation of phospholipase C-activating G-protein coupled receptor signaling pathway<br>positive regulation of protein localization to nucleus<br>positive regulation of protein phosphorylation<br>positive regulation of reactive oxygen species metabolic process<br>positive regulation of release of sequestered calcium ion into cytosol<br>regulation of blood coagulation<br>regulation of cell shape<br>regulation of cytosolic calcium ion concentration<br>regulation of gene expression<br>[illegible]

FA5_HUMAN	Coagulation factor V	F5	blood circulation<br>blood coagulation<br>COPII vesicle coating<br>ER to Golgi vesicle-mediated transport<br>platelet activation<br>platelet degranulation<br>response to vitamin K
FAM3B_HUMAN	Protein FAM3B	FAM3B	apoptotic process<br>insulin secretion
FAT4_HUMAN	Protocadherin Fat 4	FAT4	branching involved in ureteric bud morphogenesis<br>cerebral cortex development<br>condensed mesenchymal cell proliferation<br>fibroblast growth factor receptor signaling pathway<br>heart morphogenesis<br>heterophilic cell-cell adhesion via plasma membrane cell adhesion molecules<br>hippo signaling<br>homophilic cell adhesion via plasma membrane adhesion molecules<br>Notch signaling pathway<br>ossification involved in bone maturation<br>plasma membrane organization<br>regulation of metanephric nephron tubule epithelial cell differentiation

FBN1_HUMAN	Fibrillin-1	FBN1	activation of protein kinase A activity<br>camera-type eye development<br>cell adhesion mediated by integrin<br>cellular response to insulin-like growth factor stimulus<br>cellular response to transforming growth factor beta stimulus<br>extracellular matrix disassembly<br>extracellular matrix organization<br>glucose homeostasis<br>glucose metabolic process<br>heart development<br>metanephros development<br>negative regulation of osteoclast development<br>negative regulation of osteoclast differentiation<br>protein kinase A signaling<br>regulation of cellular response to growth factor stimulus<br>sequestering of BMP in extracellular matrix<br>sequestering of TGFbeta in extracellular matrix<br>skeletal system development

FIBG_HUMAN	Fibrinogen gamma chain	FGG	blood coagulation<br>blood coagulation, fibrin clot formation<br>cell-matrix adhesion<br>cellular protein complex assembly<br>cellular response to interleukin-1<br>cellular response to interleukin-6<br>extracellular matrix organization<br>fibrinolysis<br>negative regulation of endothelial cell apoptotic process<br>negative regulation of extrinsic apoptotic signaling pathway via death domain receptors<br>negative regulation of platelet aggregation<br>plasminogen activation<br>platelet aggregation<br>platelet degranulation<br>platelet maturation<br>positive regulation of ERK1 and ERK2 cascade<br>positive regulation of exocytosis<br>positive regulation of heterotypic cell-cell adhesion<br>positive regulation of peptide hormone secretion<br>positive regulation of protein secretion<br>positive regulation of substrate adhesion-dependent cell spreading<br>positive regulation of vasoconstriction<br>protein polymerization<br>protein secretion<br>response to calcium ion<br>toll-like receptor signaling pathway

VGFR3_HUMAN	Vascular endothelial growth factor receptor 3	FLT4	blood vessel morphogenesis<br>cellular response to vascular endothelial growth factor stimulus<br>lung alveolus development<br>lymphangiogenesis<br>lymph vessel development<br>negative regulation of apoptotic process<br>peptidyl-tyrosine phosphorylation<br>positive regulation of cell proliferation<br>positive regulation of endothelial cell migration<br>positive regulation of endothelial cell proliferation<br>positive regulation of ERK1 and ERK2 cascade<br>positive regulation of JNK cascade<br>positive regulation of MAPK cascade<br>positive regulation of protein kinase C signaling<br>positive regulation of protein phosphorylation<br>positive regulation of vascular endothelial growth factor production<br>protein autophosphorylation<br>regulation of blood vessel remodeling<br>respiratory system process<br>sprouting angiogenesis<br>transmembrane receptor protein tyrosine kinase signaling pathway<br>vascular endothelial growth factor receptor signaling pathway<br>vasculature development
FREM3_HUMAN	FRAS1-related extracellular matrix protein 3	FREM3	cell adhesion<br>cell communication
GNPTG_HUMAN	N-acetylglucosamine-1-phosphotransferase subunit gamma	GNPTG	carbohydrate phosphorylation<br>N-glycan processing to lysosome

GRM5_HUMAN	Metabotropic glutamate receptor 5	GRM5	adenylate cyclase-inhibiting G-protein coupled glutamate receptor signaling pathway<br>chemical synaptic transmission<br>cognition<br>G-protein coupled glutamate receptor signaling pathway<br>learning<br>locomotory behavior<br>phospholipase C-activating G-protein coupled glutamate receptor signaling pathway<br>regulation of long-term neuronal synaptic plasticity<br>regulation of synaptic transmission, glutamatergic
H2AW_HUMAN	Core histone macro-H2A.2	H2AFY2	brain development<br>chromatin silencing<br>covalent chromatin modification<br>dosage compensation<br>establishment of protein localization to chromatin<br>negative regulation of gene expression, epigenetic<br>negative regulation of transcription from RNA polymerase II promoter<br>negative regulation of transcription of nuclear large rRNA transcript from RNA polymerase I promoter<br>nucleosome assembly<br>positive regulation of keratinocyte differentiation
H2B1H_HUMAN	Histone H2B type 1-H	HIST1H2BH*	nucleosome assembly<br>protein ubiquitination
H2B1L_HUMAN	Histone H2B type 1-L	HIST1H2BL*	nucleosome assembly<br>protein ubiquitination
H2B1N_HUMAN	Histone H2B type 1-N	HIST1H2BN*	nucleosome assembly<br>protein ubiquitination
HPT_HUMAN	Haptoglobin	HP	acute-phase response<br>defense response<br>defense response to bacterium<br>negative regulation of hydrogen peroxide catabolic process<br>negative regulation of oxidoreductase activity<br>neutrophil degranulation<br>positive regulation of cell death<br>receptor-mediated endocytosis<br>response to hydrogen peroxide
HPTR_HUMAN	Haptoglobin-related protein	HPR	receptor-mediated endocytosis

HUWE1_HUMAN	E3 ubiquitin-protein ligase HUWE1	HUWE1	base-excision repair<br>cell differentiation<br>histone ubiquitination<br>neutrophil degranulation<br>positive regulation of protein targeting to mitochondrion<br>protein monoubiquitination<br>protein polyubiquitination
IGF2_HUMAN	Insulin-like growth factor II	IGF2	cellular protein metabolic process<br>glucose metabolic process<br>insulin receptor signaling pathway<br>insulin receptor signaling pathway via phosphatidylinositol 3-kinase<br>multicellular organism development<br>ossification<br>platelet degranulation<br>positive regulation of activated T cell proliferation<br>positive regulation of catalytic activity<br>positive regulation of cell division<br>positive regulation of cell proliferation<br>positive regulation of glycogen (starch) synthase activity<br>positive regulation of glycogen biosynthetic process<br>positive regulation of insulin receptor signaling pathway<br>positive regulation of MAPK cascade<br>positive regulation of mitotic nuclear division<br>positive regulation of peptidyl-tyrosine phosphorylation<br>positive regulation of protein kinase B signaling<br>positive regulation of protein phosphorylation<br>regulation of gene expression by genetic imprinting<br>regulation of transcription, DNA-templated S<br>skeletal system development

IGHG1_HUMAN	Ig gamma-1 chain C region	IGHG1	B cell receptor signaling pathway<br>complement activation<br>complement activation, classical pathway<br>defense response to bacterium<br>Fc-gamma receptor signaling pathway involved in phagocytosis<br>innate immune response<br>phagocytosis, engulfment<br>phagocytosis, recognition<br>positive regulation of B cell activation
IGHG4_HUMAN	Ig gamma-4 chain C region	IGHG4	B cell receptor signaling pathway<br>complement activation<br>complement activation, classical pathway<br>defense response to bacterium<br>Fc-gamma receptor signaling pathway involved in phagocytosis<br>innate immune response<br>phagocytosis, engulfment<br>phagocytosis, recognition<br>positive regulation of B cell activation
IGHM_HUMAN	Ig mu chain C region	IGHM	adaptive immune response<br>antibacterial humoral response<br>B cell receptor signaling pathway<br>complement activation, classical pathway<br>innate immune response<br>leukocyte migration<br>phagocytosis, engulfment<br>phagocytosis, recognition<br>positive regulation of B cell activation
IGLL5_HUMAN	Immunoglobulin lambda-like polypeptide 5	IGLL5	B cell receptor signaling pathway<br>complement activation, classical pathway<br>defense response to bacterium<br>innate immune response<br>phagocytosis, engulfment<br>phagocytosis, recognition<br>positive regulation of B cell activation

IHH_HUMAN	Indian hedgehog protein	IHH	cartilage development<br>cell-cell signaling<br>intein-mediated protein splicing<br>maternal process involved in female pregnancy<br>negative regulation of alpha-beta T cell differentiation<br>negative regulation of apoptotic process<br>negative regulation of immature T cell proliferation in thymus<br>negative regulation of signal transduction<br>negative regulation of T cell differentiation in thymus<br>positive regulation of alpha-beta T cell differentiation<br>positive regulation of chondrocyte differentiation<br>positive regulation of epithelial cell proliferatio<br>positive regulation of smoothened signaling pathway<br>positive regulation of T cell differentiation in thymus<br>positive regulation of transcription from RNA polymerase II promoter
IL25_HUMAN	Interleukin-25	IL25	eosinophil differentiation<br>inflammatory response to antigenic stimulus<br>interleukin-13 production<br>interleukin-5 production<br>positive regulation of transcription from RNA polymerase II promoter<br>response to fungus<br>response to nematode

IQGA1_HUMAN	Ras GTPase-activating-like protein IQGAP1	IQGAP1	cellular response to calcium ion<br>cellular response to epidermal growth factor stimulus<br>cellular response to platelet-derived growth factor stimulus<br>epidermal growth factor receptor signaling pathway<br>fibroblast growth factor receptor signaling pathway<br>glomerular visceral epithelial cell development<br>negative regulation of dephosphorylation<br>neuron projection extension<br>neutrophil degranulation<br>platelet-derived growth factor receptor signaling pathway<br>positive regulation of cellular protein localization<br>positive regulation of dendrite development<br>positive regulation of focal adhesion assembly<br>positive regulation of MAP kinase activity<br>positive regulation of peptidyl-tyrosine autophosphorylation<br>positive regulation of protein kinase activity<br>positive regulation of vascular associated smooth muscle cell migration<br>regulation of cytokine production<br>response to angiotensin<br>signal transduction
ITIH1_HUMAN	Inter-alpha-trypsin inhibitor heavy chain H1	ITIH1	hyaluronan metabolic process: May act as a carrier of hyaluronan in serum or as a binding protein between hyaluronan and other matrix protein, including those on cell surfaces in tissues to regulate the localization, synthesis and degradation of hyaluronan which are essential to cells undergoing biological processes
ITIH2_HUMAN	Inter-alpha-trypsin inhibitor heavy chain H2	ITIH2	hyaluronan metabolic process: May act as a carrier of hyaluronan in serum or as a binding protein between hyaluronan and other matrix protein, including those on cell surfaces in tissues to regulate the localization, synthesis and degradation of hyaluronan which are essential to cells undergoing biological processes
KIF27_HUMAN	Kinesin-like protein KIF27	KIF27	cilium assembly<br>epithelial cilium movement<br>microtubule-based movement<br>ventricular system development

KIF3B_HUMAN	Kinesin-like protein KIF3B	KIF3B	anterograde axonal transport<br>antigen processing and presentation of exogenous peptide antigen via MHC class II<br>determination of left/right symmetry<br>intraciliary transport involved in cilium assembly<br>microtubule-based movement<br>mitotic centrosome separation<br>mitotic spindle assembly<br>mitotic spindle organization<br>plus-end-directed vesicle transport along microtubule<br>positive regulation of cytokinesis<br>retrograde vesicle-mediated transport, Golgi to ER
KLK6_HUMAN	Kallikrein-6	KLK6	amyloid precursor protein metabolic process<br>central nervous system development<br>collagen catabolic process<br>hormone metabolic process<br>myelination<br>neuron death<br>positive regulation of G-protein coupled receptor protein signaling pathway<br>protein autoprocessing<br>protein processing<br>regulation of cell differentiation<br>regulation of neuron projection development<br>response to wounding<br>tissue regeneration
LAMA3_HUMAN	Laminin subunit alpha-3	LAMA3	cell adhesion<br>endodermal cell differentiation<br>epidermis development<br>extracellular matrix disassembly<br>extracellular matrix organization<br>hemidesmosome assembly<br>regulation of cell adhesion<br>regulation of cell migration<br>regulation of embryonic development

LAMB3_HUMAN	Laminin subunit beta-3	LAMB3	brown fat cell differentiation<br>cell adhesion<br>endodermal cell differentiation<br>epidermis development<br>extracellular matrix disassembly<br>extracellular matrix organization<br>hemidesmosome assembly
LFTY2_HUMAN	Left-right determination factor 2	LEFTY2	BMP signaling pathway<br>cell development<br>cell growth Source: InterPro<br>multicellular organism development<br>platelet degranulation Source: Reactome<br>positive regulation of pathway-restricted SMAD protein phosphorylation<br>regulation of apoptotic process<br>regulation of MAPK cascade<br>SMAD protein signal transduction<br>transforming growth factor beta receptor signaling pathway
LR16A_HUMAN	Leucine-rich repeat-containing protein 16A	LRRC16A; CARMIL1	actin filament network formation<br>actin filament organization<br>barbed-end actin filament uncapping<br>blood coagulation<br>cell migration<br>lamellipodium assembly<br>macropinocytosis<br>negative regulation of barbed-end actin filament capping<br>positive regulation of actin filament polymerization<br>positive regulation of cell migration<br>positive regulation of lamellipodium organization<br>positive regulation of stress fiber assembly<br>positive regulation of substrate adhesion-dependent cell spreading
LRC17_HUMAN	Leucine-rich repeat-containing protein 17	LRRC17	bone marrow development<br>negative regulation of osteoclast differentiation<br>ossification

LRRK2_HUMAN	Leucine-rich repeat serine/threonine-protein kinase 2	LRRK2	activation of MAPK activity<br>activation of MAPKK activity<br>autophagy<br>calcium-mediated signaling<br>canonical Wnt signaling pathway<br>cellular protein localization<br>cellular response to dopamine<br>cellular response to manganese ion<br>cellular response to oxidative stress<br>cellular response to starvation<br>determination of adult lifespan<br>endocytosis<br>excitatory postsynaptic potential<br>exploration behavior<br>Golgi organization<br>GTP metabolic process<br>intracellular distribution of mitochondria<br>intracellular signal transduction<br>locomotory exploration behavior<br>lysosome organization<br>MAPK cascade<br>mitochondrion localization<br>mitochondrion organization<br>negative regulation of autophagosome assembly<br>negative regulation of endoplasmic reticulum stress-induced intrinsic apoptotic signaling pathway<br>negative regulation of excitatory postsynaptic potential<br>negative regulation of GTPase activity<br>negative regulation of hydrogen peroxide-induced cell death negative regulation of late endosome to lysosome transport<br>negative regulation of macroautophagy<br>[illegible]
MUC6_HUMAN	Mucin-6	MUC6	maintenance of gastrointestinal epithelium<br>O-glycan processing<br>stimulatory C-type lectin receptor signaling pathway

NICA_HUMAN	Nicastrin	NCSTN	amyloid precursor protein catabolic process Source: HGNC<br>amyloid precursor protein metabolic process<br>beta-amyloid formation Source: UniProtKB<br>ephrin receptor signaling pathway<br>epithelial cell proliferation<br>membrane protein ectodomain proteolysis<br>membrane protein intracellular domain proteolysis<br>myeloid cell homeostasis<br>neutrophil degranulation<br>Notch receptor processing<br>Notch signaling pathway<br>positive regulation of apoptotic process<br>positive regulation of catalytic activity<br>protein processing<br>proteolysis<br>T cell proliferation
NEBL_HUMAN	Nebulette	NEBL	cardiac muscle thin filament assembly
NUCB1_HUMAN	Nucleobindin-1	NUCB1	regulation of protein targeting<br>response to cisplatin
PC11X_HUMAN	Protocadherin-11 X-linked	PCDH11X	homophilic cell adhesion via plasma membrane adhesion molecules<br>negative regulation of phosphatase activity

PCKGM_HUMAN	Phosphoenolpyruvate carboxykinase [GTP], mitochondrial	PCK2	cellular response to glucose stimulus<br>cellular response to tumor necrosis factor<br>gluconeogenesis<br>NADH oxidation<br>oxaloacetate metabolic process<br>positive regulation of insulin secretion<br>pyruvate metabolic process<br>response to dexamethasone<br>response to lipopolysaccharide
PCLO_HUMAN	Protein piccolo	PCLO	cAMP-mediated signaling<br>cytoskeleton organization<br>insulin secretion<br>presynapse to nucleus signaling pathway<br>protein localization to synapse<br>regulation of exocytosis<br>synapse assembly<br>synaptic vesicle exocytosis
PEX1_HUMAN	Peroxisome biogenesis factor 1	PEX1	microtubule-based peroxisome localization<br>peroxisome organization<br>protein import into peroxisome matrix<br>protein targeting to peroxisome
PGM2_HUMAN	Phosphoglucomutase-2	PGM2	deoxyribose phosphate catabolic process<br>galactose catabolic process<br>glucose metabolic process<br>glycogen biosynthetic process<br>glycogen catabolic process<br>neutrophil degranulation<br>pentose-phosphate shunt

PKHA1_HUMAN	Pleckstrin homology domain-containing family A member 1	PLEKHA1	androgen metabolic process<br>B cell receptor signaling pathway<br>cellular response to hydrogen peroxide<br>establishment of protein localization<br>estrogen metabolic process<br>face morphogenesis<br>Leydig cell differentiation<br>luteinization<br>multicellular organism growth<br>negative regulation of protein kinase B signaling<br>palate development<br>phosphatidylinositol 3-kinase signaling<br>phosphatidylinositol biosynthetic process<br>platelet-derived growth factor receptor signaling pathway<br>post-embryonic development
PLMN_HUMAN	Plasminogen	PLG	blood coagulation<br>cellular protein metabolic process<br>extracellular matrix disassembly<br>fibrinolysis<br>negative regulation of cell-cell adhesion mediated by cadherin<br>negative regulation of cell proliferation<br>negative regulation of cell-substrate adhesion<br>negative regulation of fibrinolysis<br>platelet degranulation<br>positive regulation of fibrinolysis<br>tissue remodeling
TRY1_HUMAN	Trypsin-1	PRSS1	cobalamin metabolic process<br>digestion<br>extracellular matrix disassembly

PSA4_HUMAN	Proteasome subunit alpha type-4	PSMA4	anaphase-promoting complex-dependent catabolic process<br>antigen processing and presentation of exogenous peptide antigen via MHC class I, TAP-dependent<br>Fc-epsilon receptor signaling pathway<br>MAPK cascade<br>negative regulation of canonical Wnt signaling pathway<br>negative regulation of G2/M transition of mitotic cell cycle<br>negative regulation of ubiquitin-protein ligase activity involved in mitotic cell cycle<br>NIK/NF-kappaB signaling<br>positive regulation of canonical Wnt signaling pathway<br>positive regulation of ubiquitin-protein ligase activity involved in regulation of mitotic cell cycle transition<br>proteasome-mediated ubiquitin-dependent protein catabolic process<br>protein deubiquitination<br>protein polyubiquitination<br>regulation of cellular amino acid metabolic process<br>regulation of mRNA stability<br>regulation of transcription from RNA polymerase II promoter in response to hypoxia<br>SCF-dependent proteasomal ubiquitin-dependent protein catabolic process<br>stimulatory C-type lectin receptor signaling pathway<br>T cell receptor signaling pathway<br>transmembrane transport<br>tumor necrosis factor-mediated signaling pathway<br>Wnt signaling pathway, planar cell polarity pathway
RBMX_HUMAN	RNA-binding motif protein, X chromosome	RBMX	cellular response to interleukin-1<br>gene expression<br>membrane protein ectodomain proteolysis<br>mRNA splice site selection<br>mRNA splicing, via spliceosome<br>negative regulation of mRNA splicing, via spliceosome<br>osteoblast differentiation<br>positive regulation of mRNA splicing, via spliceosome<br>positive regulation of transcription from RNA polymerase II promoter<br>protein homooligomerization<br>regulation of alternative mRNA splicing, via spliceosome<br>transcription from RNA polymerase II promoter

RREB1_HUMAN	Ribonucleoside-diphosphate reductase subunit M2 B	RREB1	multicellular organism development<br>negative regulation of transcription from RNA polymerase II promoter<br>positive regulation of epithelial cell migration<br>positive regulation of lamellipodium morphogenesis<br>positive regulation of mammary gland epithelial cell proliferation<br>positive regulation of substrate adhesion-dependent cell spreading<br>positive regulation of transcription, DNA-templated<br>positive regulation of transcription from RNA polymerase II promoter<br>positive regulation of wound healing, spreading of epidermal cells<br>Ras protein signal transduction<br>regulation of transcription, DNA-templated<br>transcription from RNA polymerase II promoter
RIR2B_HUMAN	Ribonucleoside-diphosphate reductase subunit M2 B	RRM2B	deoxyribonucleoside triphosphate metabolic process<br>deoxyribonucleotide biosynthetic process<br>DNA repair<br>kidney development<br>mitochondrial DNA replication<br>negative regulation of intrinsic apoptotic signaling pathway by p53 class mediator<br>nucleobase-containing small molecule interconversion<br>renal system process<br>response to amine<br>response to oxidative stress
S14L2_HUMAN	SEC14-like protein 2	SEC14L2	positive regulation of transcription, DNA-templated Source: UniProtKB<br>regulation of cholesterol biosynthetic process Source: UniProtKB<br>transcription, DNA-templated
A1AT_HUMAN	Alpha-1-antitrypsin	SERPINA1	acute-phase response<br>blood coagulation<br>COPII vesicle coating<br>ER to Golgi vesicle-mediated transport<br>neutrophil degranulation<br>platelet degranulation

SHH_HUMAN	Sonic hedgehog protein	SHH	androgen metabolic process<br>animal organ formation<br>apoptotic signaling pathway<br>artery development<br>blood coagulation<br>canonical Wnt signaling pathway<br>CD4-positive or CD8-positive, alpha-beta T cell lineage commitment<br>cell-cell signaling<br>cell development<br>cell fate specification endocytosis<br>epithelial cell proliferation involved in salivary gland morphogenesis<br>epithelial-mesenchymal signaling involved in prostate gland development<br>lymphoid progenitor cell differentiation<br>mesenchymal cell proliferation involved in lung development<br>mesenchymal smoothened signaling pathway involved in prostate gland development<br>metanephric mesenchymal cell proliferation involved in metanephros development<br>myoblast differentiation<br>myotube differentiation<br>negative regulation of alpha-beta T cell differentiation<br>negative regulation of apoptotic process<br>negative regulation of canonical Wnt signaling pathway<br>negative regulation of cell differentiation<br>negative regulation of cell migration<br>negative regulation of cholesterol efflux<br>negative regulation of dopaminergic neuron differentiation<br>negative regulation of kidney smooth muscle cell differentiation<br>negative regulation of mesenchymal cell apoptotic process<br>negative regulation of proteasomal ubiquitin-dependent protein catabolic process<br>negative regulation of T cell proliferation<br>negative regulation of transcription elongation from RNA polymerase II promoter<br>negative regulation of transcription from RNA polymerase II promoter<br>[illegible]

MPCP_HUMAN	Phosphate carrier protein, mitochondrial	SLC25A3	mitochondrial transport<br>transport
SMC2_HUMAN	Structural maintenance of chromosomes protein 2	SMC2	cell division<br>kinetochore organization<br>meiotic chromosome condensation<br>meiotic chromosome segregation<br>mitotic chromosome condensation
SNTB2_HUMAN	Beta-2-syntrophin	SNTB2	
SPTN4_HUMAN	Spectrin beta chain, non-erythrocytic 4	SPTBN4	actin filament capping<br>cytoskeletal anchoring at plasma membrane<br>ER to Golgi vesicle-mediated transport<br>establishment of protein localization to plasma membrane<br>MAPK cascade<br>positive regulation of multicellular organism growth<br>regulation of peptidyl-serine phosphorylation<br>regulation of sodium ion transport<br>vesicle-mediated transport
TRFE_HUMAN	Serotransferin	TF*	cellular iron ion homeostasis<br>cellular response to iron ion<br>ferrous iron import into cell<br>iron ion homeostasis<br>membrane organization<br>platelet degranulation<br>positive regulation of receptor-mediated endocytosis<br>regulation of protein stability<br>transferrin transport

TIMP4_HUMAN	Metalloproteinase inhibitor 4	TIMP4	central nervous system development<br>negative regulation of catalytic activity<br>negative regulation of membrane protein ectodomain proteolysis<br>Notch signaling pathway<br>ovulation cycle<br>response to cytokine<br>response to drug<br>response to hormone<br>response to lipopolysaccharide<br>response to peptide hormone
TITIN_HUMAN	Titin	TTN	mitotic chromosome condensation<br>platelet degranulation<br>regulation of catalytic activity S<br>regulation of protein kinase activity<br>response to calcium ion<br>sarcomere organization<br>sarcomerogenesis
TTHY_HUMAN	Transthyretin	TTR	cellular protein metabolic process<br>extracellular matrix organization<br>neutrophil degranulation<br>retinoid metabolic process<br>retinol metabolic process<br>thyroid hormone transport<br>transport

UB2V2_HUMAN	Ubiquitin-conjugating enzyme E2 variant 2	UBE2V2	cell proliferation<br>DNA double-strand break processing<br>double-strand break repair via nonhomologous end joining<br>error-free postreplication DNA repair<br>negative regulation of neuron apoptotic process<br>positive regulation of DNA repair<br>positive regulation of neuron projection development<br>positive regulation of proteasomal ubiquitin-dependent protein catabolic process<br>positive regulation of synapse assembly<br>postreplication repair<br>protein K63-linked ubiquitination<br>protein polyubiquitination<br>protein ubiquitination<br>regulation of DNA repair
VASH1_HUMAN	Vasohibin-1	VASH1	angiogenesis<br>cell cycle arrest<br>labyrinthine layer blood vessel development<br>negative regulation of angiogenesis<br>negative regulation of blood vessel endothelial cell migration<br>negative regulation of endothelial cell migration<br>negative regulation of endothelial cell proliferation<br>negative regulation of lymphangiogenesis<br>regulation of cellular senescence<br>response to wounding

VAV3_HUMAN	Guanine nucleotide exchange factor VAV3	VAV3	angiogenesis<br>B cell receptor signaling pathway<br>cellular response to DNA damage stimulus<br>ephrin receptor signaling pathway<br>Fc-epsilon receptor signaling pathway<br>Fc-gamma receptor signaling pathway involved in phagocytosis<br>integrin-mediated signaling pathway<br>lamellipodium assembly<br>neutrophil chemotaxis<br>platelet activation<br>positive regulation of apoptotic process<br>positive regulation of B cell proliferation<br>positive regulation of cell adhesion<br>positive regulation of phosphatidylinositol 3-kinase activity<br>regulation of cell size<br>regulation of GTPase activity<br>regulation of Rho protein signal transduction<br>regulation of small GTPase mediated signal transduction<br>response to drug<br>small GTPase mediated signal transduction<br>vascular endothelial growth factor receptor signaling pathway<br>vesicle fusion
VITRN_HUMAN	Vitrin	VIT	extracellular matrix organization<br>positive regulation of cell-substrate adhesion
WWP1_HUMAN	NEDD4-like E3 ubiquitin-protein ligase WWP1	WWP1	ion transmembrane transport Source: Reactome<br>negative regulation of transcription, DNA-templated<br>proteasome-mediated ubiquitin-dependent protein catabolic process<br>protein ubiquitination<br>signal transduction

Table S3

The 10 significant expressed proteins in the proteome with MS quantitation from 6 patient (P) and 4 donor (D)

Uniprot KB	P1	P2	P3	P4	P5	P6	D1	D2	D3	D4	average patients	averag e	median patient	median donors	ratio P/D average	ration P/D median
ACLY_HUMAN	4.24	5.23	4.81	0	3.27	6.34	0	0	0	0	3.98166667	0	4.525	0	#DIV/0!	#DIV/0!
APOD_HUMAN	0	0	0	0	0	0	8.51	8.59	8.59	9.93	0	8.905	0	8.59	0	0
ATD3A_HUMAN	5.65	0	5.54	6.82	0	5.34	8.43	6.93	5.84	6.53	3.89166667	6.933	5.44	6.73	0.56137	0.808321
BHA09_HUMAN	3.83	6.39	5.84	7.03	6.34	7.62	7.68	7.2	7.83	9.43	6.175	8.035	6.365	7.755	0.76851	0.820761
CTCFL_HUMAN	0	0	0	0	11.3	9.73	10.1	11.3	11.3	11.3	3.50333333	10.99	0	11.29	0.31892	0
CTNB1_HUMAN	0	0	0	0	0	4.25	4.77	0	4.63	11.2	0.70833333	5.16	0	4.7	0.13727	0
DCA15_HUMAN	7.53	7.25	7.02	6.11	9.86	7.84	5.82	3.91	6.34	6.15	7.60166667	5.555	7.39	5.985	1.36844	1.234754
RPOM_HUMAN	10.8	10.7	10.8	10.7	10.8	0	0	0	0	10.7	8.95833333	2.67	10.75	0	3.35518	#DIV/0!
TRFE_HUMAN	5.21	0	1.69	3.29	4.42	6.89	5.52	6.21	7.09	8.22	3.58333333	6.76	3.855	6.65	0.53008	0.579699
TTHY_HUMAN	10.3	10.3	10.3	10.3	10.3	10.3	10.3	10.2	10.3	10.2	10.2666667	10.25	10.27	10.25	1.00187	1.001952

ratio P/D average	ration P/D median
ratio P/D average	ration P/D median
over express	over express
under express	under express
0.56136555	0.80832095
0.76851276	0.8207608
0.31891974	under express
0.1372739	under express
1.36843684	1.23475355
3.35518102	over express
0.5300789	0.57969925

Figure S3

A.

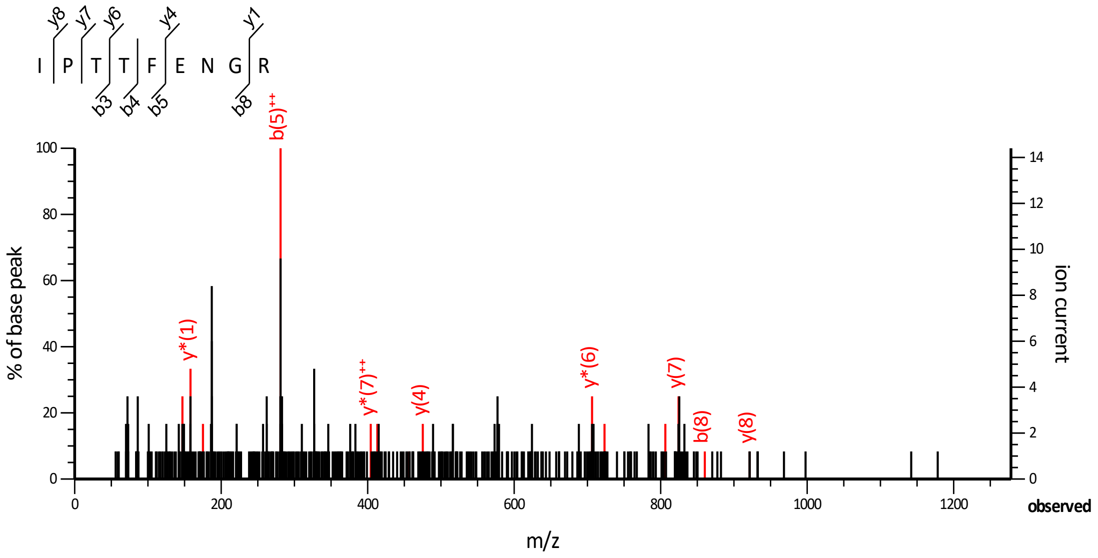

B.

```
       Tryps            Tryps   Tryps                       Tryps
         |                |       |                           |
     MFKQLSCISLPSSCDYSSRSLLQPPSPKMVMLLLLLSALAGLFGAAEGQAFHLGKCPNPP
   1 ---------+---------+---------+---------+---------+---------+   60

             Tryps                          Tryps
         Tryps   | Tryps    Tryps       Tryps |  Tryps
             |   |      |       |            | |      |
     VQENFDVNKYLGRWYEIEKIPTTFENGRCIQANYSLMENGKIKVLNQELRADGTVNQIEG
  61 ---------+---------+---------+---------+---------+---------+  120

                Tryps
            Tryps   |                                        Tryps
                |   |                                            |
     EATPVNLTEPAKLEVKFSWFMPSAPYWILATDYENYALVYSCTCIIQLFHVDFAWILARN
 121 ---------+---------+---------+---------+---------+---------+  180

                        Tryps
            Tryps    Tryps|
                |        ||
     PNLPPETVDSLKNILTSNNIDVKKMTVTDQVNCPK
 181 ---------+---------+---------+-----   215
```

Additional File 6. A MS/MS Fragmentation of apolipoprotein D, APOD (gi|619383) peptide (IPTTFENGR) from this proteome analysis (A). The protein sequence of APOD with predicted tryptic digestion site, the IPTTFENGR peptide was shown in the blue highlight pattern (B).
